# High plasticity in epithelial morphogenesis during insect dorsal closure

**DOI:** 10.1242/bio.20136072

**Published:** 2013-09-05

**Authors:** Kristen A. Panfilio, Georg Oberhofer, Siegfried Roth

**Affiliations:** 1 Institute for Developmental Biology, University of Cologne, Zülpicher Strasse 47b, 50674 Cologne, Germany; 2 J. F. Blumenbach Institute of Zoology and Anthropology, Department of Developmental Biology, Georg-August-University Göttingen, Justus-von-Liebig-Weg 11, 37077 Göttingen, Germany

**Keywords:** Insect, Dorsal closure, Extraembryonic development, Epithelial morphogenesis, Evolution of development, Serosa, Amnion, *Tribolium castaneum*

## Abstract

Insect embryos complete the outer form of the body via dorsal closure (DC) of the epidermal flanks, replacing the transient extraembryonic (EE) tissue. Cell shape changes and morphogenetic behavior are well characterized for DC in *Drosophila*, but these data represent a single species with a secondarily reduced EE component (the amnioserosa) that is not representative across the insects. Here, we examine DC in the red flour beetle, *Tribolium castaneum*, providing the first detailed, functional analysis of DC in an insect with complete EE tissues (distinct amnion and serosa). Surprisingly, we find that differences between *Drosophila* and *Tribolium* DC are not restricted to the EE tissue, but also encompass the dorsal epidermis, which differs in cellular architecture and method of final closure (zippering). We then experimentally manipulated EE tissue complement via RNAi for *Tc-zen1*, allowing us to eliminate the serosa and still examine viable DC in a system with a single EE tissue (the amnion). We find that the EE domain is particularly plastic in morphogenetic behavior and tissue structure. In contrast, embryonic features and overall kinetics are robust to *Tc-zen1^RNAi^* manipulation in *Tribolium* and conserved with a more distantly related insect, but remain substantially different from *Drosophila*. Although correct DC is essential, plasticity and regulative, compensatory capacity have permitted DC to evolve within the insects. Thus, DC does not represent a strong developmental constraint on the nature of EE development, a property that may have contributed to the reduction of the EE component in the fly lineage.

## Introduction

In the second half of embryogenesis, the insect embryo closes its body when the left and right flanks expand up to and meet at the dorsal midline, a process known as dorsal closure (DC). As the flanks advance, they replace extraembryonic (EE) tissue that had served as a provisional cover over the yolk. This process has been well studied in the fruit fly *Drosophila melanogaster* (e.g. Kiehart et al., 2000; Jacinto et al., 2002b; Hutson et al., 2003; Solon et al., 2009; Blanchard et al., 2010), where DC is a two-tissue system that involves the embryonic epidermis and the EE amnioserosa. However, the starting topography for DC is unusual in *Drosophila*, as the amnioserosa is a secondarily derived structure that never covers the embryo proper (Schmidt-Ott, 2000). The ancestral EE complement that is still found in most insects consists of a distinct serosa and amnion that cover and protect the early embryo (Panfilio, 2008). The amnion is connected to the embryo's lateral flanks, while the serosa provides a complete cover over the embryo, amnion, and yolk. Having first developed to enclose the embryo, the EE membranes later actively withdraw in a precise way prior to their elimination during DC. Throughout these morphogenetic rearrangements, the serosa and amnion are distinct players, such that DC is a three-tissue system (embryo, amnion, and serosa) in most insects.

Thus, the evolution of extraembryonic development has involved large-scale morphological changes. Such evolutionary divergence requires mechanisms to overcome developmental constraints imposed by essential processes, such as DC. We wondered what properties a three-tissue system of DC would have in comparison to *Drosophila* at different levels of biological organization (cellular, tissue, and inter-tissue), and chose the beetle *Tribolium castaneum* as a representative model for our investigations.

**Fig. 1.**
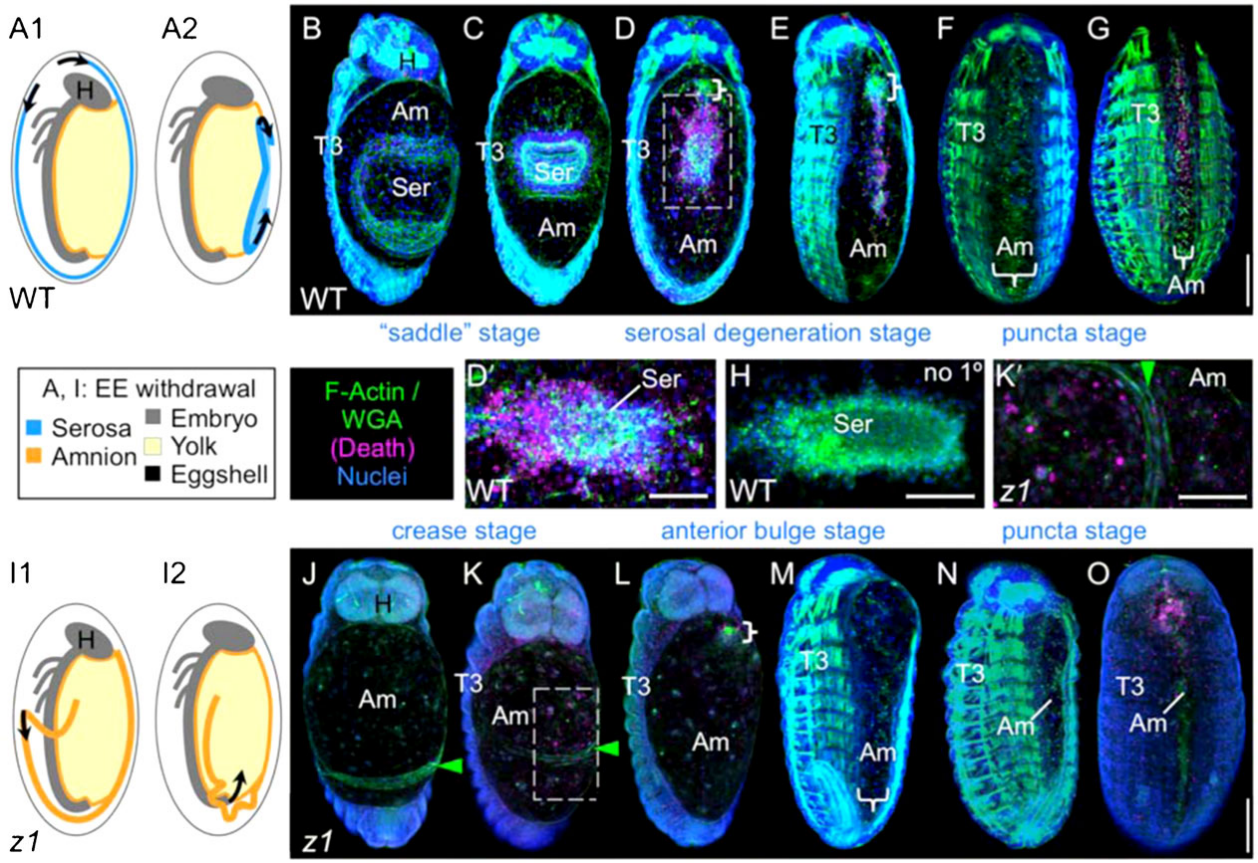
Overview of *Tribolium* DC in WT and after *Tc-zen1^RNAi^*. (A–H) WT, (I–O) *Tc-zen1^RNAi^*. (A,I) Schematics of excess EE tissue withdrawal progression: EE tissue that had covered the embryo (1) folds onto itself and contracts in the direction indicated by the arrows (2) (sagittal views, color-coding as indicated). (B–G,H,J–O) Confocal projections, dorsal views, where stage names refer to the morphology of the excess EE tissue. Whereas in WT the EE tissue is reduced to a single, planar epithelium only in late DC (F), this topography is attained earlier in *Tc-zen1^RNAi^* embryos (L). Compared to WT, the *Tc-zen1^RNAi^* amnion has higher levels of early amniotic apoptosis (K,K′), specifically in the region anterior to the tissue crease. Whole mounts are oriented with anterior up; in insets (D′,H,K′) anterior is left. Curly brackets demarcate a small, anterior ‘ball’ structure that occurs in both WT and *Tc-zen1^RNAi^* amnions (D,E,L). Green arrowheads mark the *Tc-zen1^RNAi^* amniotic crease (J,K). Fluorescent staining reagents are as indicated (see Materials and Methods): F-actin stains are shown in B–G,M,N; WGA stains are shown in H,J–L,O. Apoptosis stain (“death”) is shown in all micrographs except F,M,N, where H shows the no-primary control for the apoptosis antibody. Note that not all pycnotic nuclei are labeled with this antibody. Abbreviations: Am, amnion; H, head; Ser, serosa; T#, thoracic segment #; WT, wild type; *z1*, *Tc-zen1^RNAi^*. Scale bars: 100 µm for whole mounts (shown in G,O), 50 µm for insets. Overviews of DC morphogenesis are also provided in supplementary material Movie 1 (WT) and supplementary material Movie 5 (*Tc-zen1^RNAi^*).

As in most insects, at the beginning of late EE development, the *Tribolium* embryonic body has already shortened and thickened (undergone “germband retraction”), but it is still fully enclosed by the outer serosal membrane (Stanley and Grundmann, 1970). In wild type (WT) development, we find that EE tissue withdrawal commences with rupture of the serosa under the embryo's head, and proceeds as the serosa contracts into the center of the widest part of the back, where it thoroughly degenerates by apoptosis (Fig. 1A–E, cf. Fig. 1H; supplementary material Movie 1). In the serosa's wake is the second EE membrane, the amnion, which retains its connection to the epidermis and serves as the provisional yolk cover until the completion of DC (Fig. 1A–G). Changing amnion–serosa interactions will comprise a future study in their own right (K.A.P., unpublished). For the current investigation of DC, the starting arrangement involves morphologically distinct amniotic tissue restricted to the dorsal rim of the embryo, where it is also connected to the serosa (Fig. 1A1). It is only after serosal degeneration that the amnion alone serves as a single, planar EE epithelium, which is the starting topography of the *Drosophila* amnioserosa. However, by the time *Tribolium* has achieved this arrangement, DC is already quite advanced (Fig. 1F).

Thus, compared to the expectation of a smooth, single-tissue EE domain, the serosa represents a conspicuous structural heterogeneity during *Tribolium* DC, as it becomes a thick, folded structure with high levels of filamentous actin (F-actin) and cell death in the middle of the EE domain (Fig. 1A2–D,D′, and see below). From *Drosophila* DC studies, it has been argued that apoptosis correlates with maintenance of tension in a contractile epithelium as it reduces in apical surface area (Toyama et al., 2008; Gorfinkiel et al., 2009). Although the contracting and apoptosing features suggest that the *Tribolium* serosa could actively contribute to DC, it also comprises a relative excess of EE tissue to be eliminated, and it could equally cause a delay in DC compared to a system in which a single EE tissue participates, as in *Drosophila*.

It is here that *Tribolium* provides a key advantage as a study system for DC. It is possible to genetically remove serosal identity while maintaining larval viability – including completion of DC – via RNAi for *Tc-zen1*, which encodes a class 3 Hox transcription factor (van der Zee et al., 2005). This is a special feature of only some *zen* genes, including *Tc-zen1* (van der Zee et al., 2005; Rafiqi et al., 2008), as other *zen* genes have an entirely different role in late development (van der Zee et al., 2005; Panfilio et al., 2006), while loss of the *Drosophila zen* orthologue is lethal (Wakimoto et al., 1984). Knock down of *Tc-zen1* leads to an early respecification of presumptive serosal cells to either an embryonic or amniotic fate, thereby reducing EE tissue with respect to both cell number (as some presumptive serosa becomes embryonic) and cell identity (amnion only) (van der Zee et al., 2005). We therefore knocked down *Tc-zen1* and examined how the embryo is able to regulate for this change so as to successfully complete DC. This experimental approach enabled us to examine DC along two lines of enquiry. Firstly, comparison of WT and *Tc-zen1^RNAi^* elucidates compensatory changes that are involved in serosa-less DC, which we find is less robust than in WT. Secondly, with a single EE tissue, *Tc-zen1^RNAi^* embryos provide a study system for more direct interspecific comparison with *Drosophila*, which we nonetheless find to be quite different in several respects. As *Tribolium* DC has thus far not been described, we first characterize WT DC, and then go on to an analysis of the knockdown phenotype.

## Results

### Changing morphologies during WT tissue withdrawal in *Tribolium* distinguish the amnion and serosa

**Fig. 2.**
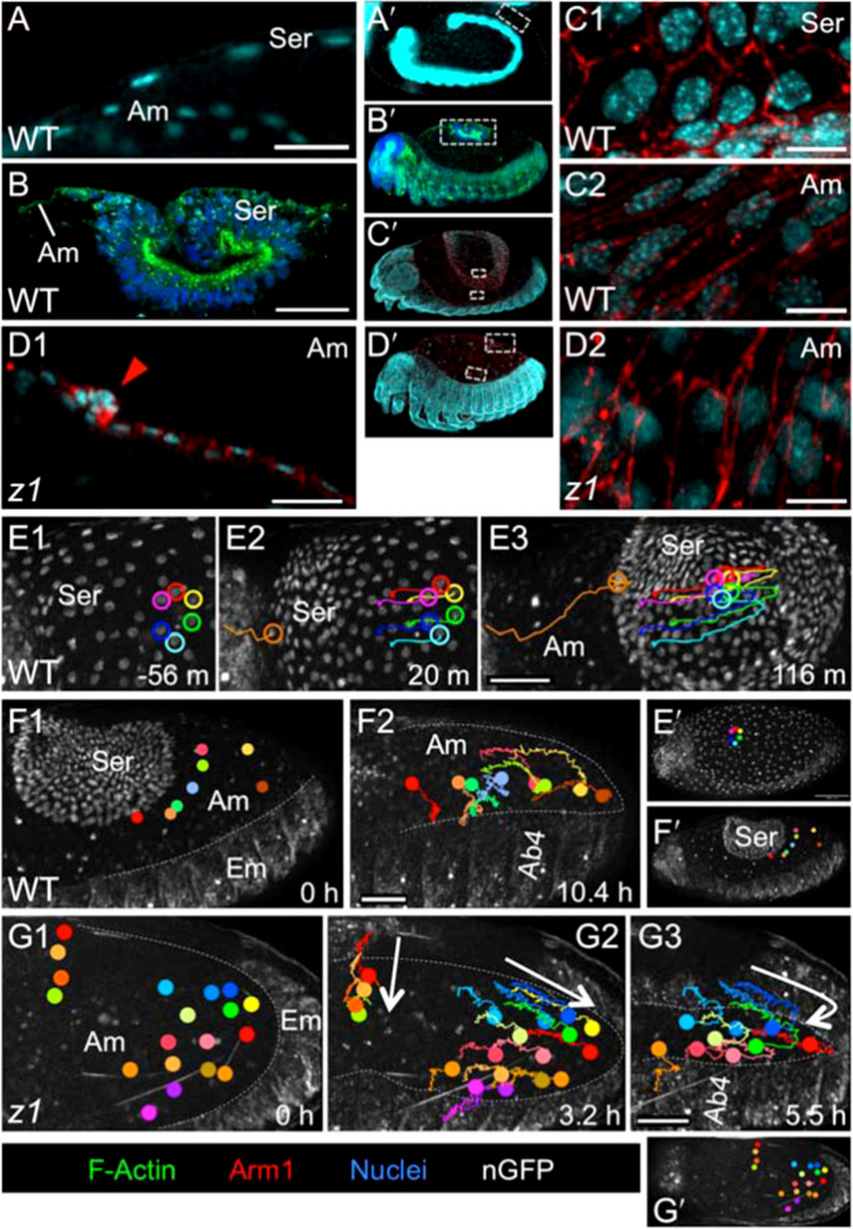
The serosa and amnion are distinguishable by cellular morphology and rearrangement behavior. (A,B,C,E,F) WT, (D,G) *Tc-zen1^RNAi^*. (A–D) Serosal cells have larger, brighter (more polyploid), more widely spaced nuclei; more hexagonal apical areas; and high levels of apical F-Actin during contraction (A–C). The *Tc-zen1^RNAi^* tissue retains amniotic characteristics (D). Stages shown before (A) and during (B,C,D) EE tissue withdrawal, in sagittal (A,B,D1) and surface (C1,C2,D2) views. The arrowhead indicates the *Tc-zen1^RNAi^* amniotic crease (D1). (E–G) Nuclear tracking from supplementary material Movies 1, 2 (E,F) and supplementary material Movies 6, 7 (G), where colored dots indicate individual tracked cells' current positions. Serosal cells more rigidly keep their neighbors during tissue reorganization than WT or *Tc-zen1^RNAi^* amniotic cells (note crossed pink and green tracks in F2), though overall amniotic cells converge toward the dorsal midline during DC (tracks in F2,G2,G3). See supplementary material Fig. S1 for quantification. Elapsed time is relative to serosal rupture (E) or the first panel shown (F,G). F1 starts 3.9 hours after E1 (same embryo). Dashed lines approximate the amnion–embryo border (F,G). White arrows indicate general trends of tissue reorganization (G2,G3). In whole embryo views (letter-prime panels), regions of interest are indicated by dashed grey boxes or colored dots (E1/F1/G1 and E′/F′/G′ are of the same time point). Images are oriented with anterior left and dorsal up. Visualization reagents are as indicated (B also stained with WGA: cyan). Abbreviations as in previous figure; additionally: Ab#, abdominal segment #; Em, embryo; m, minutes; h, hours. Scale bars: 20 µm (A,D1), 50 µm (B,E1–E3,F1,F2,G1–G3), and 10 µm (C1,C2,D2).

Serosal withdrawal involves a wholesale reorganization: a tissue that had encompassed the entire egg volume compacts into a small structure known as the dorsal organ (i.e. the “saddle” stage, Fig. 1A2–C), with a ∼15-fold reduction in surface area. What had been a monolayered, squamous epithelial sheet with widely spaced nuclei (Fig. 2A) becomes a structure composed of tall, wedge-shaped, pseudostratified cells, which folds over onto itself and maintains high levels of apical F-actin (Fig. 2B).

In contrast, the smaller amniotic cells, with much smaller nuclei, remain squamous throughout development (Fig. 2A,B). Serosal and amniotic cells are readily distinguished throughout these events by their nuclei: the larger, brighter appearance of the serosal nuclei is consistent with their high degree of polyploidy (Fig. 2A,C1,C2) (Truckenbrodt, 1973). This difference is also apparent with a transgenic line that ubiquitously expresses nuclear-GFP (nGFP) (Sarrazin et al., 2012): the large serosal and closely packed epidermal nuclei are much more readily detected than the amniotic nuclei (Fig. 2E–F). The two EE tissues also differ in cellular rearrangements (supplementary material Movie 2). Whereas serosal cells strictly keep their neighbors within the tissue sheet during withdrawal (Fig. 2E1–E3), amniotic cells reorganize relative to one another as they converge toward the midline during DC (Fig. 2F1,F2, quantified in supplementary material Fig. S1). This is part of a general convergent extension of the entire dorsum during DC due to ventral flexure of the embryo (supplementary material Movie 1; Fig. 6I), which is associated with head lobe morphogenesis (Stanley and Grundmann, 1970; Posnien et al., 2010).

**Fig. 3.**
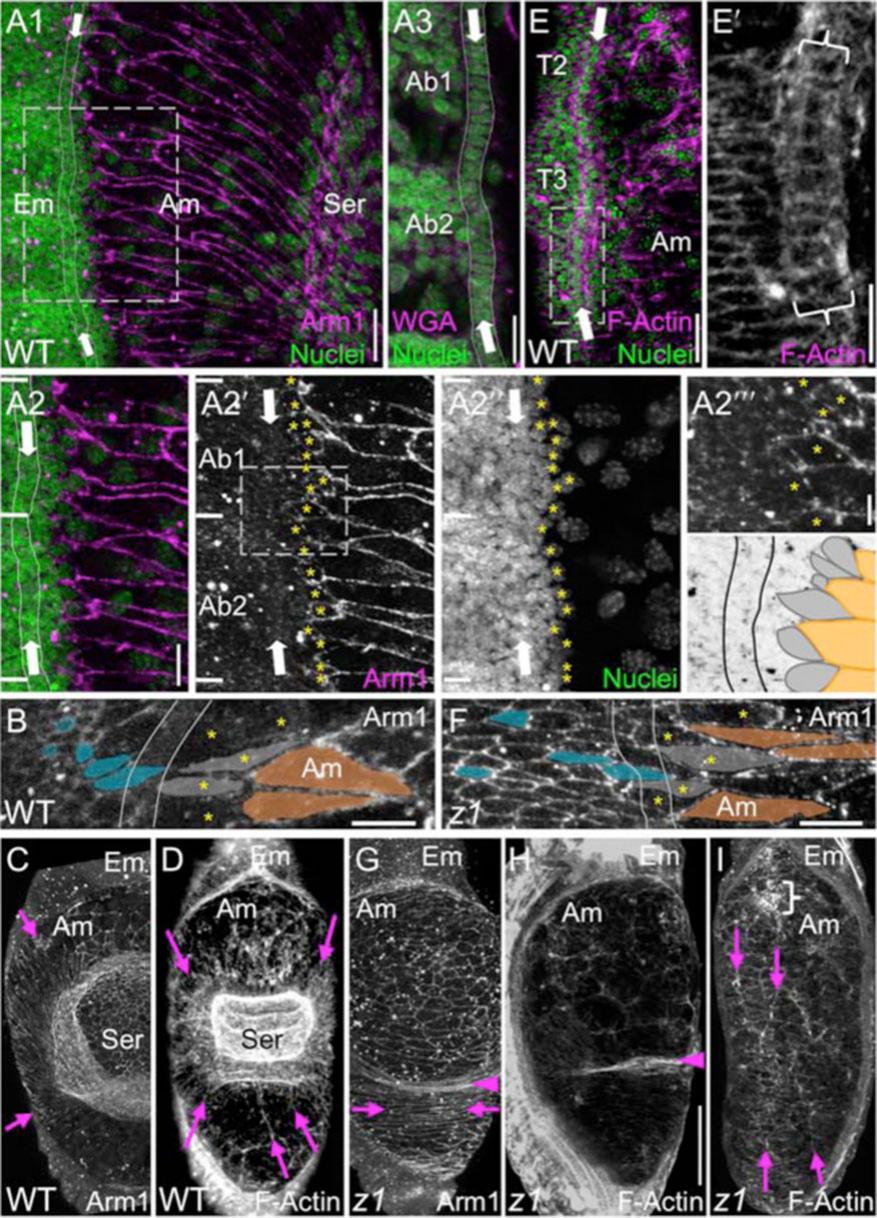
Tissue structure during DC in the epidermis and amnion. (A–E) WT, (F–I) *Tc-zen1^RNAi^*. (A–I) Confocal projections of early (A,C,D,G,H), and mid (B,E,F,I) DC illustrate several shared features between WT and *Tc-zen1^RNAi^* DC: the straggling organization of the dorsal-most epidermal cells (A1,A2–A2‴,B,F: starred cells), the more lateral F-actin-enriched but unpolarized cardioblast cell row (A3 [partial projection], E,E′: arrows, curly brackets), and amniotic cell elongation around the serosa or amniotic crease (A1,A2,C,G: arrows). In contrast, amniotic F-actin fiber organization differs (D,I: arrows). For comparison, the subsurface cardioblast cell row is superimposed in A1,A2,B,F (paired thin lines). Dashed boxes in A1,A2′,E show enlargements in A2–A3,A2‴,E′, respectively. Horizontal white lines in A2 demarcate segmental boundaries. In the A2‴ schematic and B,F, indicative cells are colored for the lateral epidermis (cyan), dorsal-most epidermis (grey), and amnion (orange). Greater epidermal cell elongation in F than B reflects a slightly older embryo, not a phenotypic difference. The *Tc-zen1^RNAi^* amniotic crease is labeled with arrowheads (G,H). The curly bracket in I marks the anterior ball structure (see also Fig. 1L). Images are oriented with anterior up and dorsal/medial right. Staining reagents are as indicated (Arm1 is anti-Tc-Arm1). For additional *Tc-zen1^RNAi^* images, see supplementary material Fig. S2. Abbreviations as in previous figures. Panels C and D show the same two embryos as in Fig. 2C and Fig. 1C, respectively. Scale bars: 20 µm (A,E), 10 µm (A2–A2″,B,E′,F), 5 µm (A2‴), and 100 µm (shown in H for C,D,G–I).

Such individual rearrangements of amniotic cells are limited in earlier DC. Cell shapes throughout the period of serosal withdrawal (saddle and serosal degeneration stages, Fig. 1B–E) seem in part determined by their limited intra-tissue mobility whilst experiencing extrinsic tension due to serosal contraction. Amniotic cells adjacent to the contracting serosa show remarkable elongation in the medial–lateral direction (M–L, also referred to as the dorsal–ventral axis, D–V), in contrast to more distant amniotic cells or the epidermis (Fig. 3A1,A2–A2″,B,C). Furthermore, the amnion displays a radial array of F-actin fibers around the serosa (Fig. 3D), which is also consistent with it being pulled upon by the latter tissue. However, the exact structural nature of the relationship between the amnion and the serosa is difficult to visualize at these stages, in part due to the rapidly changing outer shape of the folding serosa. In longitudinal sections, the thin amnion appears to extend up to and be in contact with the outer part of the curling serosal edges (Fig. 2B, Fig. 6B), but it is unclear whether a stable point of connection exists or the serosa is sliding past a surrounding amnion. In either case, the serosa exerts force on the amnion as it subsequently sinks down into the yolk, in the midst of the amnion, during degeneration (Fig. 1D,E; supplementary material Movie 3). Either the serosa directly tugs on the connected amniotic edges, or the serosa would have to physically make a hole in order to go through the amnion into the yolk. After serosal degeneration, all amniotic cells become transversely elongated, and ultimately characterized by small puncta of F-actin as they apically constrict out of the plane of the dorsum and die (puncta stage, Fig. 1F,G, Fig. 5A–B).

### The epidermal–amniotic boundary is irregular, with no clear leading edge

The *Tribolium* epidermis has an indistinct, straggling border of teardrop-shaped cells that are primarily distinguished by size from the adjacent, larger amniotic cells (Fig. 3A1–A2‴,B). These cells show some elongation roughly in the direction of DC progression (greater length than width aspect ratio: mean of 2.3±0.6 standard deviation, for 18 cells starred in Fig. 3A2′). However, the cells' long axes are only roughly aligned with the dorsal–ventral axis (mean angle of deviation of 22°, range of angles of 53°, where parallel to the D–V axis is 0°). Although irregular in arrangement, these dorsal-most epidermal cells in *Tribolium* differ from more lateral epidermal cells, which present smaller and rounder apical surfaces (Fig. 3A2,A2′,A2‴,B, grey *vs* blue cells, respectively).

Despite the lack of an organized LE, the *Tribolium* embryo–amnion boundary appears distinct in low magnification views (Fig. 1), due to the proximity of other embryonic structures. Chief among these is a single cell row that spans the length of the flank, with a medial supracellular actin cable (Fig. 3A3,E,E′). However, it is set back from the dorsal-most epidermal cells, also has a lateral actin cable, shows limited M–L elongation, and is subepidermal (Fig. 3A3,E′; supplementary material Fig. S2), suggesting a mesodermal origin and probable identification as the cardioblast precursors to the insect heart (the dorsal vessel) (Rugendorff et al., 1994; Reim and Frasch, 2005). Nonetheless, as DC progresses the longitudinal F-actin fibers of this structure become readily apparent in surface views (Fig. 3E, Fig. 5A–B, arrows; supplementary material Fig. S2). Given the lack of any prominent, organized embryonic structure that is more medial and epidermal, we use the cardioblast cell row as an anatomical landmark feature during DC.

### Whole system behavior involves rhythmic pulses and scalloped zippering progression

**Fig. 4.**
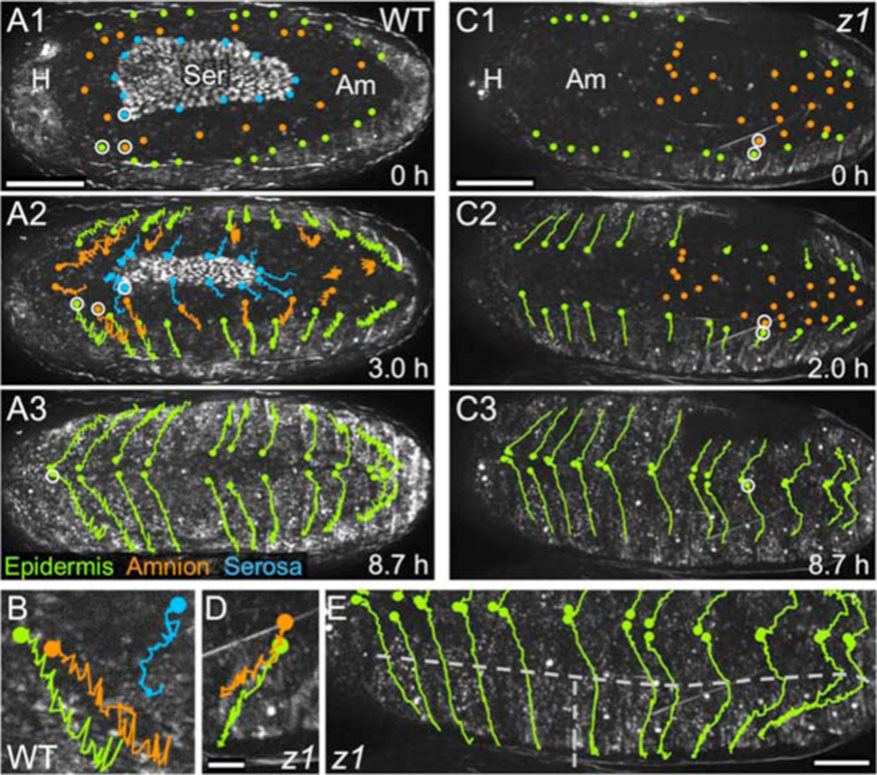
Tissue movements during both WT and *Tc-zen1^RNAi^* DC include early pulsatile progression and flank spreading, followed by a global anteriorward shift. (A–B) WT, (C–E) *Tc-zen1^RNAi^*. (A,C) Representative images from nuclear tracking analyses, with color-coding for tissue type as indicated (see also supplementary material Movies 3, 4, 6, 8). Images are stage-matched for EE area, meaning that only the later, faster phase is shown for *Tc-zen1^RNAi^* DC. For clarity, *Tc-zen1^RNAi^* amniotic tracks are omitted in C2 (but shown in supplementary material Movie 8, as are anterior nuclei that could only be followed prior to anterior bulge resorption). Shorter tracks for three individual *Tc-zen1^RNAi^* epidermal cells are due to a small, external visual obstruction. (B,D) Higher magnification of selected tracks (5.2-hour duration, earlier start time in D than in C1) that are marked with white circles in A,C, respectively. The initial zigzag track shape shows the undulating waves of DC phase I, which is more pronounced in WT. (E) Labeling of the left flank from C3, illustrating the changing epidermal cell trajectories (15.5-hour interval, starting 6.8 hours before C1): vertical dashed line demarcates anterior from posterior halves, as determined by spreading of the flanks over the egg equator; horizontal dashed line demarcates early (lower) and later (upper) track segments at the point when the anteriorward postural shift begins. This also occurs in WT (A3). Views are dorsal, with anterior left. Abbreviations as in previous figures. Scale bars: 100 µm (A1–A3,C1–C3), 20 µm (shown in D for B,D), 50 µm (E).

At the global, inter-tissue level there are three behavioral phases to *Tribolium* DC: (1) propagating posterior-to-anterior waves passing through the dorsum, (2) a fast phase when the epidermal flanks smoothly extend toward the midline, and (3) a final, slow phase during which the ‘seam’ between the flanks remains apparent (supplementary material Movies 3, 4; Fig. 4A). In the first phase, propagating waves result in a back-and-forth pattern to tracks of epidermal and amniotic cells, although the contracting serosa follows its own program of folding up into the dorsal organ (Fig. 4B). Early progression also includes a general spreading of the flanks with respect to the A–P axis as they encompass the widest part of the dorsum (i.e. extend over the “equator” of the egg: Fig. 4A2,E). Later, all cells show a slight anteriorward shift, consistent with the aforementioned embryonic postural change (Fig. 4A3).

**Fig. 5.**
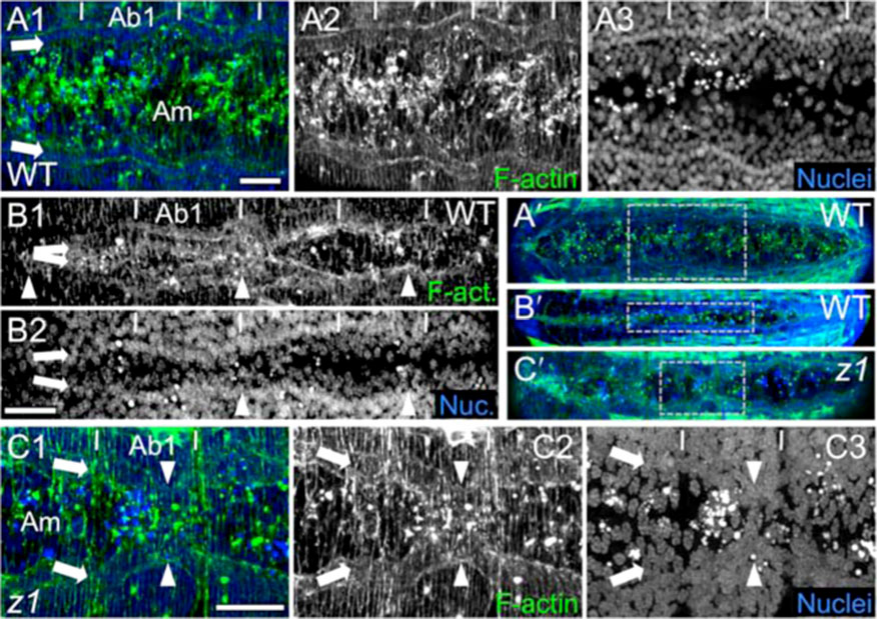
Final epidermal zippering during DC occurs at multiple independent points along the A–P axis in both WT and after *Tc-zen1^RNAi^*. (A–B) WT, (C) *Tc-zen1^RNAi^*. (A–C) Confocal projections of late DC, illustrating the transverse F-Actin fibers of the epidermis and the amniotic midline puncta of apical F-Actin and pycnotic (bright, condensed) nuclei (A,C), and final DC occurring simultaneously at several positions along the A–P axis (B,C). Two successive DC stages are shown for WT (A,B), while all features are shown in the single *Tc-zen1^RNAi^* embryo (C). See supplementary material Table S1 for numerical characterization of these features. Labeling indicates: segmental boundaries (vertical lines), the cardioblast cell rows (arrows), and scalloped maxima (arrowheads). The subsurface cardioblasts are less apparent in C3 due to projection of a thinner confocal stack than in A,B. In whole dorsum views (letter-prime panels), regions of interest are indicated by dashed grey boxes. All views are dorsal, with anterior left. Abbreviations as in previous figures. Scale bars: 20 µm.

Midline closure of the *Tribolium* epidermis occurs at multiple points independently along the A–P axis, producing a scalloped pattern (Fig. 5A–B). Here referred to as “scalloped maxima”, these regions meet at the midline in advance of adjacent tissue. There is symmetry in closure morphogenesis between the flanks, with scalloped maxima mostly occurring in left–right pairs. However, most embryos also exhibit one or two unpaired scalloped maxima. The paired maxima are roughly in segmental register, but deviations from this trend (0–3 per segment) were also observed, and there is no consistent A–P positioning of the maxima relative to segmental boundaries. Due to scalloping, the epidermal edge is longer than if it were truly straight or bowed out in a smooth curve along the dorsum (supplementary material Table S1 quantifies all of these features).

Having thus characterized WT *Tribolium* DC, in which the withdrawal of the serosa is the dominant feature until late in the process, we then examined how this integrated, multi-tissue system is altered by absence of the serosa after *Tc-zen1^RNAi^*.

### The Tc-zen1^RNAi^ EE tissue is amniotic only, but has a late developmental program that is reminiscent of WT

Although *Tc-zen1^RNAi^* embryos have no serosa, the amnion is still able to partially cover the embryo with a posterior fold of tissue that arises early in development (Fig. 1I1) (Handel et al., 2000; van der Zee et al., 2005). This posterior EE fold has consequences for DC, as it must also be eliminated. In a manner highly reminiscent of WT serosal withdrawal, the *Tc-zen1^RNAi^* posterior amniotic fold pulls up dorsally to form a crease in the tissue (Fig. 1I2–K). This transverse crease then migrates anteriorly as the back end of a protruding bulge that displaces underlying yolk (Fig. 1L,M), though ultimately the bulge is smoothed (puncta stage: Fig. 1N,O; supplementary material Movies 5, 6). Compared to the WT serosa, the *Tc-zen1^RNAi^* posterior amniotic fold comprises a modest excess of EE tissue beyond that which directly covers the yolk (compare Fig. 1B,C and Fig. 1J,K). It is also eliminated earlier during DC (compare Fig. 1F and Fig. 1L). Thus, although some EE folding morphogenesis also occurs during early *Tc-zen1^RNAi^* DC, this experimental treatment does reduce the amount of EE tissue to a situation more comparable to *Drosophila*.

Furthermore, the *Tc-zen1^RNAi^* EE tissue retains the characteristics of the WT amnion. Specifically, the cells remain small and squamous, with small nuclei with relatively weak fluorescence (Fig. 2D1,D2,G). This tissue also exhibits the same degree of intra-tissue cell rearrangement as in the WT amnion, as cells change neighbors (Fig. 2G1–G3, e.g. posterior red and green tracks; supplementary material Movie 7; quantified in supplementary material Fig. S1). Finally, just as the early *Tc-zen1^RNAi^* embryo was shown to express the amniotic marker *pannier* (*GATAx*) throughout the EE tissue (van der Zee et al., 2005), we detect no serosal markers in the late stages investigated here (data not shown; few early patterning genes maintain EE expression at these stages, including *pannier*). Thus, *Tc-zen1^RNAi^* DC does represent a system with a single EE tissue type, the amnion.

### The epidermis is robust to the effects of Tc-zen1^RNAi^ on DC

Despite the EE alterations, the *Tc-zen1^RNAi^* epidermis retains WT features, including the indistinct leading edge, lack of M–L elongation, and the proximity of the subepidermal cardioblast cell row (Fig. 3F; supplementary material Fig. S2). We also find that global behavior and the manner of final epidermal closure are largely unaffected. *Tc-zen1^RNAi^* embryos exhibit the same three kinetic phases, though at slightly different morphological stages. In WT, the propagating waves (phase I) are apparent as soon as the serosa has fully contracted onto the dorsal surface, and continue throughout the saddle and serosal degeneration stages, only subsiding when serosal degeneration is far advanced (Fig. 4A–B; supplementary material Movie 3). In *Tc-zen1^RNAi^* embryos, the propagating waves commence later, once the excess tissue is largely confined to the anterior bulge and the EE dorsum is primarily a smooth plane of tissue (Fig. 4C–D; supplementary material Movies 6, 8). Nonetheless, the degree of left–right symmetry, initial A–P spreading of the flanks, and later anteriorward shift of the dorsum still occur (Fig. 4C1–C3,E). Likewise, for final zippering we did not observe a difference in degree of scalloping between WT and *Tc-zen1^RNAi^* DC (Fig. 5C; supplementary material Table S1). However, for morphologically stage-matched embryos, the distance between scalloped maxima is greater in the anterior and mid dorsum regions in *Tc-zen1^RNAi^* embryos, due to the anterior bulge (supplementary material Table S1).

### Tc-zen1^RNAi^ affects the amnion's apoptosis profile and cytoskeletal organization

In the EE domain, amniotic cell shapes during early DC are also comparable in WT and *Tc-zen1^RNAi^* embryos. Cells surrounding the contracting excess EE tissue – the WT serosa and the *Tc-zen1^RNAi^* amniotic crease – display similarly elongated shapes, while more anterior amniotic cells have rounder apical areas (compare Fig. 3C and Fig. 3G). However, in two respects the *Tc-zen1^RNAi^* amnion displays novel arrangements.

Firstly, the *Tc-zen1^RNAi^* amnion has higher levels of apoptosis in early DC (compare Fig. 1C–E and Fig. 1K), specifically in the bulge region anterior to the crease (Fig. 1K′). Later, amniotic apoptosis persists at higher levels in the region with more EE material to be eliminated (Fig. 1O).

After the elimination of the serosa, all WT amniotic cells exhibit transverse (M–L) elongation, both of overall cell shape and of the orientation of F-actin fibers (Fig. 5A1–A3,B1,B2). Thus, one might expect a transverse F-actin arrangement across the amnion throughout *Tc-zen1^RNAi^* DC, reflecting a uniform EE tissue under tension and/or exerting contractile force across the dorsum. Instead, there are no notable F-actin structures in the *Tc-zen1^RNAi^* amnion during early DC except for the crease (Fig. 3H, cf. Fig. 3D). Later, the tissue is rather characterized by supracellular F-actin fibers oriented roughly parallel to the closing embryonic flanks (Fig. 3I). It is only in late *Tc-zen1^RNAi^* DC that the remaining amniotic region acquires a transversely oriented F-actin arrangement comparable to WT (compare Fig. 5C1–C3 and Fig. 5A–B).

### Altered morphogenesis after Tc-zen1^RNAi^ has consequences for the geometry and final success of DC

**Fig. 6.**
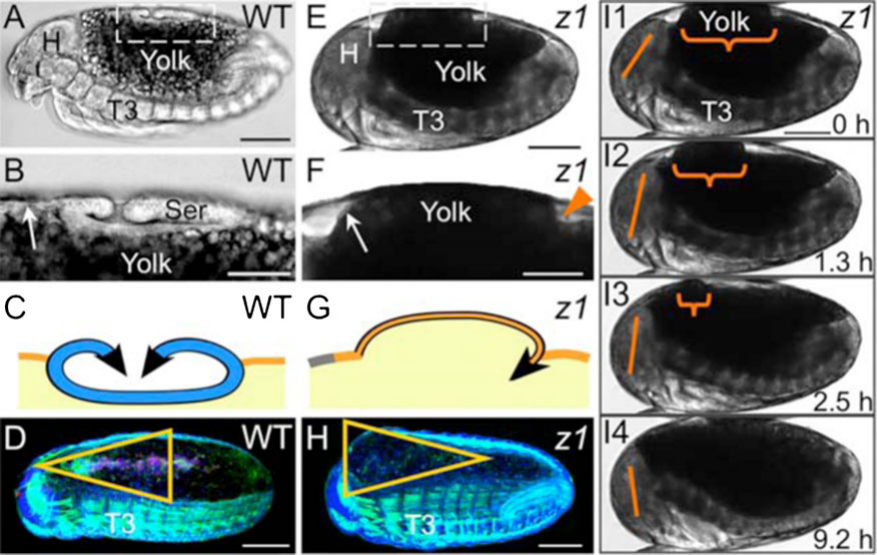
The apical–basal orientation of EE tissue contraction affects DC geometry. (A–D) WT, (E–I) *Tc-zen1^RNAi^*. (A,E) Whole embryo lateral views (transmitted light micrographs; fixed, A; live, E). (B,F) Enlargements of boxed regions in A,E. Unlike the folded serosa, the thin amnion (arrows) can hardly be distinguished from the underlying yolk. The arrowhead marks the *Tc-zen1^RNAi^* amniotic crease. For cell shape data, see Fig. 2B,D1. (C,G) Corresponding midsagittal schematics: serosal apical contraction produces a hollow structure, whereas basal *Tc-zen1^RNAi^* amniotic contraction inappropriately displaces yolk (color coding as in Fig. 1A, black for emphasis only here). (D,H) Subsequently, the overall geometry of the closing EE area is affected (triangle orientation; shown in dorsal–lateral aspect, reproduced from Fig. 1E,M). See supplementary material Table S1 for quantification. (I1–I4) Still images from supplementary material Movie 9: the yolk bulge (curly bracket) is eventually incorporated anteriorly, concomitant with an embryonic flexure that stretches the dorsum (note changing angle of the straight line between head landmarks). This postural change also occurs in WT (supplementary material Movie 1). Anterior is left and dorsal up in all panels. Abbreviations as in previous figures. Scale bars: 100 µm (A,D,E,H,I), 50 µm (B,F).

Opposite orientations of EE tissue contraction between WT and *Tc-zen1^RNAi^* embryos has larger scale consequences on DC geometry (Fig. 6). The WT serosa curls outward and folds over on itself (Fig. 6A–C), contracting apically throughout its degeneration and thus creating a hollow disk (Fig. 6B,C, Fig. 2B). In contrast, a main reason that *Tc-zen1^RNAi^* embryos have an amniotic bulge is because the inward/basal orientation of the amniotic crease's contractile force displaces underlying yolk (Fig. 6E–G).

*Tc-zen1^RNAi^* DC also differs in the site towards which EE tissue contracts. Unlike the central location of the late WT serosa (Fig. 1C), the amniotic bulge actively migrates anteriorly, even prior to the anterior shift associated with the embryonic postural change (Fig. 6I1–I4; supplementary material Movies 5, 6). Ultimately, the bulge is smoothed through active reorganization of the amnion, the aforementioned increased levels of apoptosis, and the ventral body flexure (Fig. 6I1–I4; supplementary material Movie 9). Globally, this results in a difference in shape of the closing EE area (Fig. 6D,H).

The amnion lacks the cell size and epithelial cohesion of the serosa (Fig. 2), and we find that *Tc-zen1^RNAi^* DC is less robust than in WT. The anterior bulge is particularly fragile (note slight anterior handling damage in Fig. 1M). The occasional failure of *Tc-zen1^RNAi^* DC is associated with lesions in this region, corroborated by live imaging of some *Tc-zen1^RNAi^* embryos extruding yolk dorsally, ranging from small amounts to catastrophic hemorrhaging (supplementary material Fig. S3). Furthermore, although the excess EE tissue is eliminated early relative to DC (Fig. 1L, cf. Fig. 1F), the overall process is slower, and ultimately *Tc-zen1^RNAi^* embryos complete embryogenesis later than WT embryos (morphological age scoring during late EE morphogenesis, and hatch rate after DC: from 64–68 to 74–78 hours, *n* = 329 WT, 72 *Tc-zen1^RNAi^*).

## Discussion

### Tc-zen1^RNAi^ amniotic regulation enables DC, but less effectively than in WT with two EE tissues

We have analyzed dorsal closure in the beetle *Tribolium* for cell, tissue, and whole system features in wild type and after extraembryonic tissue reduction via *Tc-zen1^RNAi^*. Our nuclear tracking analyses and examination of cell shapes in fixed tissues (with the recently described nGFP transgenic line (Sarrazin et al., 2012) and the new anti-Tc-Arm1 antibody described here) provide an overview of dynamics at the tissue and whole-system levels. In future analyses, additional live imaging tools for cell membranes and the actin cytoskeleton (Benton et al., 2013) will be valuable for examining amniotic epithelial reorganization and the mode of extension (Tada and Heisenberg, 2012) of the dorsum in detail.

Interestingly, a number of features are robust to EE manipulation via *Tc-zen1^RNAi^*, including the structure of the epidermis (dorsal-most cell shape and organization, simultaneous zippering at multiple points along the length of the dorsum: Fig. 3A,E, Fig. 4; supplementary material Fig. S2) and the overall behavior of the system (ventral embryonic flexure; successive DC phases of rhythmic undulations, smooth advance, persistent seam: Fig. 4; supplementary material Movies 3, 6). Furthermore, the *Tc-zen1^RNAi^* amnion retains WT amnion characteristics: small nuclear and cell size, squamous cell shape, and extreme cell elongation near to morphogenetically active EE tissue amid a tissue that otherwise lacks early supracellular F-actin structures (Fig. 2D, Fig. 3G,H).

**Fig. 7.**
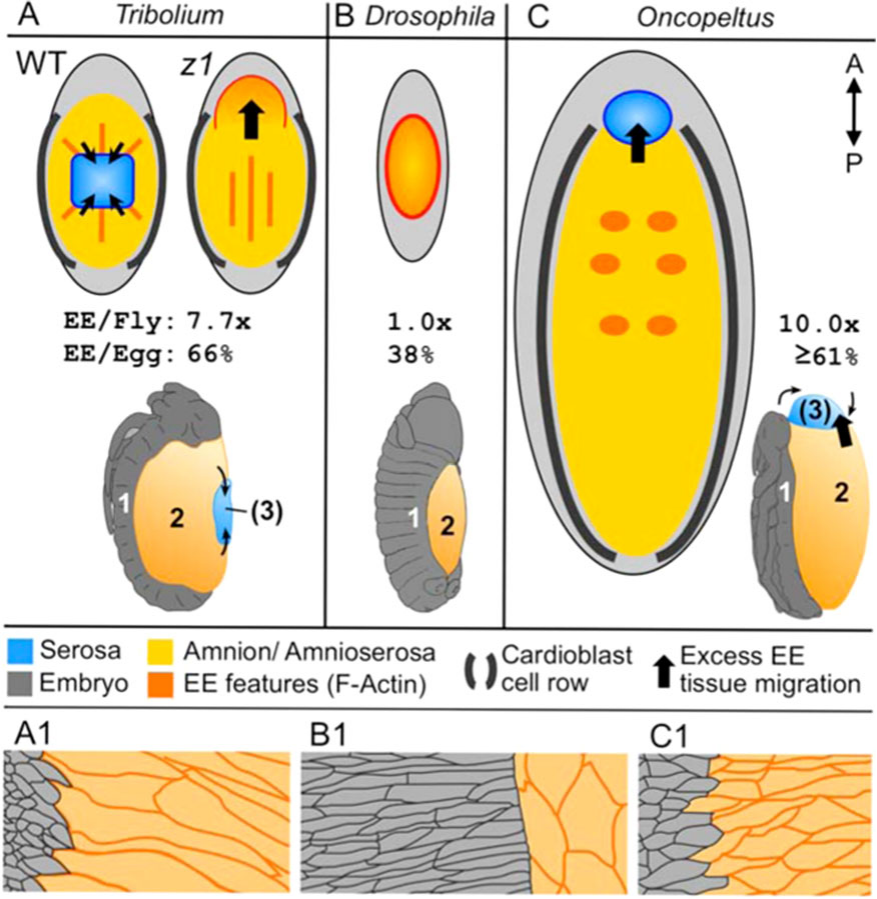
Interspecific comparison of DC for morphological features and dorsum size. (A) *Tribolium* (WT and *Tc-zen1^RNAi^* (*z1*)), (B) *Drosophila,* and (C) *Oncopeltus* are shown at early-mid DC. Upper schematics represent dorsal views, shown to scale, with relative EE areas compared to *Drosophila*'s (“EE/Fly”) and to total dorsal area (“EE/Egg”) given below (*n*≧3 per species). The ≧ designation for *Oncopeltus* reflects the limited availability of the youngest, widest DC stages for measurement. Prominent EE features are shown: *Tribolium* radial (WT) or longitudinal (*Tc-zen1^RNAi^*) F-actin fibers and anterior bulge, *Drosophila* radial contractility gradient from the leading edge F-actin cable, *Oncopeltus* bilateral thoracic clusters. Lower schematics, in lateral aspect, indicate the number of tissues participating in DC (adapted from Campos-Ortega and Hartenstein, 1997 (*Drosophila*) and Panfilio, 2008 (*Oncopeltus*)). (A1–C1) Apical areas of cells at the epidermal–extraembryonic boundary, shown for approximately three epidermal and two amniotic/amnioserosal cell rows, emphasizing the epidermal cell elongation and regularity of the tissue boundary in *Drosophila*, in contrast to the amniotic elongation and irregular border in *Tribolium* and *Oncopeltus*. Schematized representations are based on: anti-Tc-Arm1 (Fig. 3A), alpha-Catenin-GFP (Jacinto et al., 2002b), and phalloidin (Panfilio and Roth, 2010), respectively.

However, the mechanism of *Tc-zen1^RNAi^* DC is distinct from WT in several ways (Fig. 7A). There is no detectable serosal EE tissue after *Tc-zen1^RNAi^* (Figs 1, 2; supplementary material Fig. S1). Although the amount of excess amnion (Fig. 1I1–J) was greater than what we had anticipated from the previous characterization of younger developmental stages (van der Zee et al., 2005), it is eliminated earlier than the WT serosa (Fig. 1E,L). Nonetheless, the *Tc-zen1^RNAi^* amniotic crease results from the early presence of excess amnion, and this structure does affect subsequent DC progression.

The *Tc-zen1^RNAi^* amniotic crease's morphogenetic behavior (basalward contraction displacing the yolk dorsal-anteriorly) differs from that of the WT serosa (apical contraction in the center of the dorsum). Although it is only a minor fold (Fig. 2D1), the crease is enriched in F-actin (Fig. 3H), and the anterior bulge of displaced yolk is associated with the crease's contractility (Fig. 6; supplementary material Movie 9). Consistent with this, the minority of *Tc-zen1^RNAi^* embryos that fail to complete DC (supplementary material Fig. S3) shows loss of amniotic structural integrity when the yolk bulge breaks through (stages in Fig. 1M–O). The anterior amnion's susceptibility to tearing under strain may be because it is thin and limited in cell number. Although the topographical context differs, there is indirect evidence that too little amnion impairs DC in the fly *Megaselia abdita* after u-shaped group gene knockdown, and that augmenting amniotic cell number via *Ma-zen^RNAi^* actually improves the embryo's ability to perform DC in this context (Rafiqi et al., 2010). Future biomechanical analyses will help to clarify the nature of the forces produced during *Tc-zen1^RNAi^* DC.

Meanwhile, in our current study we have generated a system with a different mode of DC, and we find that WT DC is more rapid and robust than *Tc-zen1^RNAi^* DC. Although a *Tc-zen1^RNAi^* system with no excess amnion may be still more efficient, our data are also consistent with an interpretation in which serosal contraction and degeneration promote DC, which we discuss further in the context of interspecific comparisons, below. Furthermore, in the absence of the serosa the *Tc-zen1^RNAi^* amnion shows novel modifications that may facilitate DC: apoptosis occurs where excess tissue must be eliminated (Fig. 1K,O), and the longitudinal supracellular F-actin fibers (Fig. 3I) may indicate an enhanced role for amniotic cell rearrangement. As the serosa does appear to affect DC and the amnion shows potentially relevant morphogenetic innovations not observed in WT, we conclude that *Tc-zen1^RNAi^* DC represents a case of developmental regulation.

### Many differences in DC between the beetle and the fly

Overall, *Tribolium* DC is very different from *Drosophila* DC. Whereas the amnion–epidermal boundary is irregular in *Tribolium* (Fig. 7A1), *Drosophila* has a clearly delineated leading edge (LE) row of highly polarized epidermal cells, which show clear elongation in the direction of migration (mean of 7-fold greater D–V length than A–P width, *n* = 16 cells) and have pointed ventral edges (Jacinto et al., 2002b) (depicted in Fig. 7B1). This orientation is the opposite of the pointed dorsal/medial edge of *Tribolium*'s less elongated, teardrop shaped cells (aspect ratio of 2.3, Fig. 3A2′). The supracellular actin cable at the medial edge of *Drosophila* LE cells is a hallmark of *Drosophila* DC (Edwards et al., 1997; Kiehart et al., 2000; Jacinto et al., 2002a; Solon et al., 2009), whereas in *Tribolium* the only notable F-actin cables in the vicinity are those on both the medial and lateral sides of the underlying cardioblast cell row. As for the EE tissue, amnioserosal cells shorten on the D–V/M–L axis (Blanchard et al., 2009) (Fig. 7B1), whereas in *Tribolium* it is the amniotic cells that undergo M–L elongation.

Regarding specialization within the EE domain, although the fly amnioserosa exhibits several subtle A–P and radial asymmetries (Peralta et al., 2007; Toyama et al., 2008; Gorfinkiel et al., 2009; Solon et al., 2009), it is quite homogeneous compared to the beetle amnion (radial or longitudinal F-actin arrays), much less compared to a WT system with a centrally contracting serosa surrounded by an amnion. Even in the one EE tissue system of both WT *Drosophila* and *Tc-zen1^RNAi^* DC, EE cellular rearrangements differ substantially. Amnioserosal cell neighbor exchange via intercalation is limited (Blanchard et al., 2009) and most cell loss occurs progressively from the periphery (Sokolow et al., 2012). Indeed, the extent to which the amnioserosal cells retain their neighbors (Pope and Harris, 2008) is more akin to what we observe in the WT *Tribolium* serosa (Fig. 2E). In contrast, *Tribolium* amniotic cells converge toward the dorsal midline (Fig. 2F,G; supplementary material Fig. S1), and cell alignment (Fig. 3I) suggests that there could be polarity within the plane of the amnion that contributes to DC (Nishimura et al., 2007; St Johnston and Sanson, 2011). However, in both species the widest EE region in early DC exhibits a higher rate of apoptosis (anterior amnioserosa in the fly (Toyama et al., 2008; Gorfinkiel et al., 2009); *Tribolium*, Fig. 1D,K), supporting a role for local EE apoptosis in contributing to tissue tension during DC (Toyama et al., 2008). With respect to whole system behavior, individual amnioserosal cells pulse but do not participate in larger-scale tissue movements (Solon et al., 2009), in contrast to the rhythmic pulses across the dorsum in *Tribolium* early DC (Fig. 4; supplementary material Movies 3, 6).

Lastly, epidermal zippering differs between *Tribolium* and *Drosophila*. In contrast to the scalloped approach in *Tribolium* (Fig. 5), *Drosophila* DC proceeds via progressive zippering from the outer corners (“canthi”) toward the center of the back (Kiehart et al., 2000). Intriguingly, paired scalloped maxima can be generated in *Drosophila* after mechanical or genetic abrogation of the leading edge actin cable, with ectopic expression of the cell adhesion molecule Echinoid resulting in a *Drosophila* epidermal organization highly reminiscent of that in WT *Tribolium* (Hutson et al., 2003; Peralta et al., 2007; Laplante and Nilson, 2011). In other words, given the opportunity, scalloped closure occurs in *Drosophila* when the flanks are in close proximity. This implies that the actual difference is that a relatively more rapid rate of central flank closure in *Tribolium* provides this opportunity in normal development.

### Broader interspecific comparison of insect DC, and its potential evolvability

Several features of DC in *Tribolium* that differ from *Drosophila* are also found in the milkweed bug, *Oncopeltus fasciatus*, which we had previously described (Panfilio, 2009; Panfilio and Roth, 2010). *Tribolium* and *Drosophila* are holometabolous insects, which undergo complete metamorphosis before the adult stage. *Oncopeltus* belongs to the more ancestral lineage of hemimetabolous insects, which lack complete metamorphosis. Hemimetabolous insects also have the full complement of both serosa and amnion, but during WT DC the amnion alone can be directly compared with the *Drosophila* amnioserosa, as the serosa degenerates in the anterior (Fig. 7C) (Panfilio, 2008). Like *Tribolium*, *Oncopeltus* DC is characterized by an indistinct leading edge (Fig. 7C1), a prominent cardioblast cell row (referred to as a “racing stripe” in this study), and the same three phases of global kinetics (Panfilio, 2009; Panfilio and Roth, 2010). The similar cell shapes on either side of the epidermal–amniotic border in these two species suggests that the very different morphology seen in *Drosophila* (Fig. 7A1–C1) could be the product of a subsequent evolutionary innovation for greater differentiation between these two tissues.

It is intriguing that both the *Tc-zen1^RNAi^* excess amnion and WT *Oncopeltus* serosa migrate anteriorly (Fig. 7A,C, thick black arrows). However, the *Tc-zen1^RNAi^* amnion is not directly comparable to the WT *Oncopeltus* serosa or amnion. Firstly, the *Tc-zen1^RNAi^* amnion does not undergo the characteristic squamous-to-columnar cell shape changes of a serosa (Fig. 2G1, cf. Fig. 2B) (Panfilio and Roth, 2010). Secondly, the *Oncopeltus* amnion is characterized by transverse cell elongation and F-actin fiber orientation throughout DC (Panfilio and Roth, 2010), unlike the longitudinal F-actin arrangement in *Tc-zen1^RNAi^* DC (Fig. 3I). Thirdly, *Oncopeltus* DC involves extensive amniotic regionalization, with spatio-temporally precise apoptosis and F-actin enrichment (Fig. 7C) (Panfilio and Roth, 2010). In contrast, early amniotic apoptosis in *Tc-zen1^RNAi^* embryos is only roughly localized and more limited in extent (Fig. 1K, anterior to the crease), and alterations to the F-actin organization affect the entire tissue (Fig. 3I). Thus, different EE tissues in different species exhibit a diversity of morphogenetic rearrangements at the cell and tissue levels.

As the EE surface area to be closed during DC is an order of magnitude larger in *Oncopeltus* than in *Drosophila* (Fig. 7), we previously posited that such EE regionalization reflected mechanical need: the regions of increased apoptosis and F-actin correlate with faster closure of the widest part of the dorsum and consequential straightening of the epidermal flanks (Panfilio and Roth, 2010). Wild type DC in the relatively large and broad eggs of *Tribolium* (Fig. 7A) provide further support for this idea, as higher levels of central-dorsal apoptosis (WT serosal degeneration) also correlate with a faster rate of flank closure in the middle of the body (Fig. 1B–E), which is not achieved after *Tc-zen1^RNAi^* (compare Fig. 1E and Fig. 1M; observations of WT and *Tc-zen1^RNAi^* egg aspect ratio (Jacobs et al., 2013)). In this interpretation, the *Tribolium* serosa represents a productive regionalization of the EE domain that promotes DC. We also suggested that the F-actin-enriched cardioblast cell row may serve as a support structure (Panfilio and Roth, 2010). It would be interesting to examine whether eliminating the cardioblasts' actin cables impairs DC in these species. In contrast, *Drosophila* DC is not characterized by marked specializations within the EE domain and the prominent embryonic F-actin fibers are part of the highly structured epidermal leading edge cell row. It may be that *Drosophila* DC represents a streamlined approach possible in a small egg with a narrower EE area, but that this approach may not be scalable.

More generally, *Tribolium* late EE development seems conserved compared to classical embryological accounts. This is particularly true for serosal folding and degeneration (i.e. dorsal organ formation: Patten, 1884; Hirschler, 1909; Strindberg, 1915; Mellanby, 1936; Jung, 1966; Stanley and Grundmann, 1970; Pétavy, 1975; Enslee and Riddiford, 1981). Our *Tc-zen1^RNAi^* data suggest that this conservation may be indicative of mechanical efficacy in removing the serosa with minimum disruption to surrounding EE tissue integrity and to the yolk. The alternative approach employed by the *Tc-zen1^RNAi^* amnion (Fig. 6) is not nearly so robust, and there may be few other morphogenetic solutions possible for eliminating the serosa. Indeed, this conservation is seen in the rather different topographies of hemimetabolous and holometabolous dorsal organs (Fig. 7A,C). Another widely conserved feature is the undulating waves from posterior to anterior across the yolk and dorsum during early DC (Mahr, 1960; Cobben, 1968; Panfilio and Roth, 2010), although its significance is unclear. In contrast, the initial, rapid process of serosal rupture and withdrawal in the Holometabola has scarcely been described, having been missed in *Tribolium* histological series (Stanley and Grundmann, 1970), with an unspecified site of serosal rupture in other Holometabola (Strindberg, 1915), and with interspecific variability in wholesale execution (Hirschler, 1909; Blunck, 1914; Tiegs and Murray, 1938; Anderson, 1972). However, preliminary data with modern imaging tools suggest that our primary account in *Tribolium* may be widely applicable in the Holometabola, including in fly species that still retain a distinct serosa and amnion (García-Solache et al., 2010).

As for DC itself, our *Tribolium* data – WT and *Tc-zen1^RNAi^* – show that although all insects perform DC, there is high plasticity in how. For example, although the epidermal flanks have a similarly straightened, parallel geometry as they near the dorsal midline in both *Oncopeltus* and *Tribolium* (e.g. Fig. 1G), *Tribolium* closure is scalloped while *Oncopeltus* zippering is more akin to that in *Drosophila*, primarily progressing from the posterior canthus (Panfilio and Roth, 2010). However, as the fly LE also scallops opportunistically when the flanks are straightened (see above), *Oncopeltus* is the unique one in this three-way comparison. A broader comparison across the insect phylogeny and for egg size can be expected to reveal still further innovations. DC may be quite different in species where it is concomitant with anteriorward growth of the entire embryo over the yolk (Blunck, 1914; Rakshpal, 1962; Pétavy, 1975). Moreover, the fact that *Tribolium* can regulate for the loss of the serosa shows that DC need not represent a late developmental constraint on EE development. This precondition may have permitted the evolutionary changes in early EE specification within the fly lineage (Goltsev et al., 2007; Rafiqi et al., 2008; Schmidt-Ott et al., 2010) that have culminated in the extreme reduction represented by the *Drosophila* amnioserosa.

## Materials and Methods

### *Tribolium* stocks and RNAi

*Tribolium castaneum* (Herbst 1797) strains used were San Bernardino wild type (Brown et al., 2009) and nuclear-GFP (Sarrazin et al., 2012), maintained under normal culturing conditions (Brown et al., 2009) at 30°C, 40% RH. All experiments were performed with both.

Parental RNAi was performed by injecting pupal or young adult females with *Tc-zen1* dsRNA (1.2 or 2 µg/µl, 862 bp fragment), as described previously (van der Zee et al., 2005; Brown et al., 2009). Controls for RNAi specificity included injection of *Tc-Toll1* (Nunes da Fonseca et al., 2008), and injection of dsRNA for the empty vector multiple cloning site (224 bp, pBluescript SK+, Stratagene). The *Tc-zen1^RNAi^* phenotype (van der Zee et al., 2005) can be distinguished at all embryonic stages from the differentiated blastoderm stage onward (by tissue morphology, egg shape, and the lack of serosal cuticle (alters optical and mechanical properties of the eggshell)).

### Immunohistochemistry

Immunohistochemistry was performed as previously described (Panfilio and Roth, 2010), with the following: apoptosis marker (rabbit anti-cleaved Caspase-3, 1:40, Cell Signaling Technology; anti-rabbit-AlexaFluor-555 or -568, 1:500, Invitrogen), F-actin (phalloidin-fluorescein, 1:50, Invitrogen), cell and nuclear outlines (wheat germ agglutinin-AlexaFluor-488, 1:50, Invitrogen), nuclei (TOTO-3 iodide, 1:1000, Invitrogen; DAPI within Vectashield mountant, Vector Laboratories; or fuchsin after (Wigand et al., 1998)). Additionally, a custom antibody against the C-terminal 15 amino acids of Tc-Armadillo1 (Tc-Arm1: GenBank Accession XR_043141 (Shah et al., 2011; Bao et al., 2012)) was used as an adherens junctions marker (produced by Eurogentec, rabbit, 1:10,000, secondary detection as above). Further details on anti-Tc-Arm1, including immunoprecipitation applications, are available upon request.

Embryos were dechorionated in bleach, rinsed in tap water, fixed in 5% formaldehyde/PBS:heptane for 2–3 hours at room temperature (RT), hand dissected under PBS, and postfixed in 5% formaldehyde/PBS for 20 minutes at RT. Images were acquired with laser scanning confocals (Zeiss LSM 700, Olympus FV1000, Leica SP2).

### Live imaging and image processing

Time-lapse imaging was performed with a Zeiss Axioplan2, Zeiss AxioImager.Z2 with Apotome.2 structured illumination, Applied Precision DeltaVision with post-acquisition deconvolution, and a Zeiss LSM710 scanning confocal microscope, with brightfield, DIC, and/or EGFP-wavelength settings (Carl Zeiss, Jena, Germany; Applied Precision, Issaquah, Washington, USA). Embryos were prepared by dechorionation with bleach, rinsing with tap water, and mounting in hanging drop preparations of Halocarbon oil 700 (Sigma). Filming was performed at RT (21–25°C) or in incubators (25–32°C). Acquisition settings are provided for individual movies in supplementary material Table S2. All embryos were scored after filming to assess subsequent survival. Post-acquisition data handling was done in ImageJ software (NIH), using a photobleach correction plug-in as necessary (J. Rietdorf, Bleach Correction macro for ImageJ, 2005 (corr_bleach050405): EMBL Heidelberg, http://www.embl.de/eamnet/html/bleach_correction.html). Cell tracking was performed with the MTrackJ plug-in (v. 1.5.0; Meijering et al., 2012). All analyses were performed on ≧3 each of WT and *Tc-zen1^RNAi^* embryos.

## Supplementary Material

Supplementary Material

## References

[b1] Anderson D. T. (1972). The development of holometabolous insects. Developmental Systems: Insects, Vol. 1 Counce S J Waddington C H, edLondon: Academic Press.

[b2] Bao R. Fischer T. Bolognesi R. Brown S. J. Friedrich M. (2012). Parallel duplication and partial subfunctionalization of β-catenin/armadillo during insect evolution. Mol. Biol. Evol. 29, 647–662 10.1093/molbev/msr219 21890476 PMC3283115

[b3] Benton M. A. Akam M. Pavlopoulos A. (2013). Cell and tissue dynamics during *Tribolium* embryogenesis revealed by versatile fluorescence labeling approaches. Development 140, 3210–3220 10.1242/dev.096271 23861059 PMC3930475

[b4] Blanchard G. B. Kabla A. J. Schultz N. L. Butler L. C. Sanson B. Gorfinkiel N. Mahadevan L. Adams R. J. (2009). Tissue tectonics: morphogenetic strain rates, cell shape change and intercalation. Nat. Methods 6, 458–464 10.1038/nmeth.1327 19412170 PMC4894466

[b5] Blanchard G. B. Murugesu S. Adams R. J. Martinez-Arias A. Gorfinkiel N. (2010). Cytoskeletal dynamics and supracellular organisation of cell shape fluctuations during dorsal closure. Development 137, 2743–2752 10.1242/dev.045872 20663818

[b6] Blunck H. (1914). Die Entwicklung des *Dytiscus marginalis* L. vom Ei bis zur Imago. Zeitschrift für wissenschaftliche Zoologie 111, 76–151.

[b7] Brown S. J. Shippy T. D. Miller S. Bolognesi R. Beeman R. W. Lorenzen M. D. Bucher G. Wimmer E. A. Klingler M. (2009). The red flour beetle, *Tribolium castaneum* (Coleoptera): a model for studies of development and pest biology. Cold Spring Harb. Protoc. 2009, pdb.emo126 10.1101/pdb.emo126 20147228

[b8] Campos-Ortega J. A. Hartenstein V. (1997). The Embryonic Development Of Drosophila Melanogaster Berlin: Springer.

[b9] Cobben R. H. (1968). Evolutionary Trends In Heteroptera. Part I. Eggs, Architecture Of The Shell, Gross Embryology And Eclosion Wageningen: Centre for Agricultural Publishing and Documentation.

[b10] Edwards K. A. Demsky M. Montague R. A. Weymouth N. Kiehart D. P. (1997). GFP-moesin illuminates actin cytoskeleton dynamics in living tissue and demonstrates cell shape changes during morphogenesis in *Drosophila*. Dev. Biol. 191, 103–117 10.1006/dbio.1997.8707 9356175

[b11] Enslee E. C. Riddiford L. M. (1981). Blastokinesis in embryos of the bug, *Pyrrhocoris apterus*. A light and electron microscopic study 1. Normal blastokinesis. J. Embryol. Exp. Morphol. 61, 35–49. 7264550

[b12] García-Solache M. Jaeger J. Akam M. (2010). A systematic analysis of the gap gene system in the moth midge *Clogmia albipunctata*. Dev. Biol. 344, 306–318 10.1016/j.ydbio.2010.04.019 20433825

[b13] Goltsev Y. Fuse N. Frasch M. Zinzen R. P. Lanzaro G. Levine M. (2007). Evolution of the dorsal–ventral patterning network in the mosquito, *Anopheles gambiae*. Development 134, 2415–2424 10.1242/dev.02863 17522157

[b14] Gorfinkiel N. Blanchard G. B. Adams R. J. Martinez Arias A. (2009). Mechanical control of global cell behaviour during dorsal closure in *Drosophila*. Development 136, 1889–1898 10.1242/dev.030866 19403661 PMC2680111

[b15] Handel K. Grünfelder C. G. Roth S. Sander K. (2000). *Tribolium* embryogenesis: a SEM study of cell shapes and movements from blastoderm to serosal closure. Dev. Genes Evol. 210, 167–179 10.1007/s004270050301 11180819

[b16] Hirschler J. (1909). Die Embryonalentwicklung von *Donacia crassipes* L. *Zeitschrift für wissenschaftliche Zoologie* 92, 627–744.

[b17] Hutson M. S. Tokutake Y. Chang M.-S. Bloor J. W. Venakides S. Kiehart D. P. Edwards G. S. (2003). Forces for morphogenesis investigated with laser microsurgery and quantitative modeling. Science 300, 145–149 10.1126/science.1079552 12574496

[b18] Jacinto A. Wood W. Woolner S. Hiley C. Turner L. Wilson C. Martinez-Arias A. Martin P. (2002a). Dynamic analysis of actin cable function during *Drosophila* dorsal closure. Curr. Biol. 12, 1245–1250 10.1016/S0960-9822(02)00955-7 12176336

[b19] Jacinto A. Woolner S. Martin P. (2002b). Dynamic analysis of dorsal closure in *Drosophila*: from genetics to cell biology. Dev. Cell 3, 9–19 10.1016/S1534-5807(02)00208-3 12110163

[b20] Jacobs C. G. C. Rezende G. L. Lamers G. E. M. van der Zee M. (2013). The extraembryonic serosa protects the insect egg against desiccation. Proc. R. Soc. B 280, 20131082 10.1098/rspb.2013.1082 PMC3712428 23782888

[b21] Jung E. (1966). Untersuchungen am Ei des Speisebohnenkäfers *Bruchidius obtectus* Say (Coleoptera). I. Mitteilung: Entwicklungsgeschichtliche Ergebnisse zur Kennzeichnung des Eitypus. Zeitschrift für Morphologie und Ökologie der Tiere 56, 444–480 10.1007/BF00442293

[b22] Kiehart D. P. Galbraith C. G. Edwards K. A. Rickoll W. L. Montague R. A. (2000). Multiple forces contribute to cell sheet morphogenesis for dorsal closure in *Drosophila*. J. Cell Biol. 149, 471–490 10.1083/jcb.149.2.471 10769037 PMC2175161

[b23] Laplante C. Nilson L. A. (2011). Asymmetric distribution of Echinoid defines the epidermal leading edge during *Drosophila* dorsal closure. J. Cell Biol. 192, 335–348 10.1083/jcb.201009022 21263031 PMC3172166

[b24] Mahr E. (1960). Normale Entwicklung, Pseudofurchung und die Bedeutung des Furchungszentrums im Ei des Heimchens (*Gryllus domesticus*). Zeitschrift für Morphologie und Ökologie der Tiere 49, 263–311 10.1007/BF00424702

[b25] Meijering E. Dzyubachyk O. Smal I. (2012). Chapter 9: Methods for cell and particle tracking. Imaging And Spectroscopic Analysis Of Living Cells. Optical And Spectroscopic Techniques (Methods In Enzymology, Vol. 504) Conn P M, edSan Diego, CA: Academic Press. 10.1016/B978-0-12-391857-4.00009-4 22264535

[b26] Mellanby H. (1936). The later embryology of *Rhodnius prolixus*. Q. J. Microsc. Sci. 79, 1–42.

[b27] Nishimura M. Inoue Y. Hayashi S. (2007). A wave of EGFR signaling determines cell alignment and intercalation in the *Drosophila* tracheal placode. Development 134, 4273–4282 10.1242/dev.010397 17978004

[b28] Nunes da Fonseca R. von Levetzow C. Kalscheuer P. Basal A. van der Zee M. Roth S. (2008). Self-regulatory circuits in dorsoventral axis formation of the short-germ beetle *Tribolium castaneum*. Dev. Cell 14, 605–615 10.1016/j.devcel.2008.02.011 18410735

[b29] Panfilio K. A. (2008). Extraembryonic development in insects and the acrobatics of blastokinesis. Dev. Biol. 313, 471–491 10.1016/j.ydbio.2007.11.004 18082679

[b30] Panfilio K. A. (2009). Late extraembryonic morphogenesis and its *zen*(*RNAi*)-induced failure in the milkweed bug *Oncopeltus fasciatus*. Dev. Biol. 333, 297–311 10.1016/j.ydbio.2009.06.036 19580800

[b31] Panfilio K. A. Roth S. (2010). Epithelial reorganization events during late extraembryonic development in a hemimetabolous insect. Dev. Biol. 340, 100–115 10.1016/j.ydbio.2009.12.034 20045678

[b32] Panfilio K. A. Liu P. Z. Akam M. Kaufman T. C. (2006). *Oncopeltus fasciatus zen* is essential for serosal tissue function in katatrepsis. Dev. Biol. 292, 226–243 10.1016/j.ydbio.2005.12.028 16460723

[b33] Patten W. (1884). The development of Phryganids, with a preliminary note on the development of *Blatta germanica*. Q. J. Microsc. Sci. 24, 549–602.

[b34] Peralta X. G. Toyama Y. Hutson M. S. Montague R. Venakides S. Kiehart D. P. Edwards G. S. (2007). Upregulation of forces and morphogenic asymmetries in dorsal closure during *Drosophila* development. Biophys. J. 92, 2583–2596 10.1529/biophysj.106.094110 17218455 PMC1864829

[b35] Pétavy G. (1975). Involution des annexes embryonnaires dans l'oeuf de *Locusta migratoria migratorioïdes* R. et F. (Orthoptera: Acrididae): Morphologie et histologie. Int. J. Insect Morphol. Embryol. 4, 1–22 10.1016/0020-7322(75)90002-1

[b36] Pope K. L. Harris T. J. C. (2008). Control of cell flattening and junctional remodeling during squamous epithelial morphogenesis in *Drosophila*. Development 135, 2227–2238 10.1242/dev.019802 18508861

[b37] Posnien N. Schinko J. B. Kittelmann S. Bucher G. (2010). Genetics, development and composition of the insect head – a beetle's view. Arthropod Struct. Dev. 39, 399–410 10.1016/j.asd.2010.08.002 20800703

[b38] Rafiqi A. M. Lemke S. Ferguson S. Stauber M. Schmidt-Ott U. (2008). Evolutionary origin of the amnioserosa in cyclorrhaphan flies correlates with spatial and temporal expression changes of *zen*. Proc. Natl. Acad. Sci. USA 105, 234–239 10.1073/pnas.0709145105 18172205 PMC2224192

[b39] Rafiqi A. M. Lemke S. Schmidt-Ott U. (2010). Postgastrular *zen* expression is required to develop distinct amniotic and serosal epithelia in the scuttle fly *Megaselia*. Dev. Biol. 341, 282–290 10.1016/j.ydbio.2010.01.040 20144604

[b40] Rakshpal R. (1962). Morphogenesis and embryonic membranes of *Gryllus assimilis* (Fabricius) (Orthoptera: Gryllidae). Proceedings of the Royal Entomological Society of London. Series A, General Entomology 37, 1–12 10.1111/j.1365-3032.1962.tb00281.x

[b41] Reim I. Frasch M. (2005). The Dorsocross T-box genes are key components of the regulatory network controlling early cardiogenesis in *Drosophila*. Development 132, 4911–4925 10.1242/dev.02077 16221729

[b42] Rugendorff A. Younossi-Hartenstein A. Hartenstein V. (1994). Embryonic origin and differentiation of the *Drosophila *heart. Roux Arch. Dev. Biol. 203, 266–280 10.1007/BF00360522 28305624

[b43] Sarrazin A. F. Peel A. D. Averof M. (2012). A segmentation clock with two-segment periodicity in insects. Science 336, 338–341 10.1126/science.1218256 22403177

[b44] Schmidt-Ott U. (2000). The amnioserosa is an apomorphic character of cyclorrhaphan flies. Dev. Genes Evol. 210, 373–376 10.1007/s004270000068 11180843

[b45] Schmidt-Ott U. Rafiqi A. M. Lemke S. (2010). *Hox3/zen* and the evolution of extraembryonic epithelia in insects. Hox Genes: Studies From The 20th To The 21st Century Deutsch J S, edAustin, TX: Landes Bioscience,

[b46] Shah M. V. Namigai E. K. O. Suzuki Y. (2011). The role of canonical Wnt signaling in leg regeneration and metamorphosis in the red flour beetle *Tribolium castaneum*. Mech. Dev. 128, 342–358 10.1016/j.mod.2011.07.001 21801833

[b47] Sokolow A. Toyama Y. Kiehart D. P. Edwards G. S. (2012). Cell ingression and apical shape oscillations during dorsal closure in *Drosophila*. Biophys. J. 102, 969–979 10.1016/j.bpj.2012.01.027 22404919 PMC3296024

[b48] Solon J. Kaya-Copur A. Colombelli J. Brunner D. (2009). Pulsed forces timed by a ratchet-like mechanism drive directed tissue movement during dorsal closure. Cell 137, 1331–1342 10.1016/j.cell.2009.03.050 19563762

[b49] St Johnston D. Sanson B. (2011). Epithelial polarity and morphogenesis. Curr. Opin. Cell Biol. 23, 540–546 10.1016/j.ceb.2011.07.005 21807488

[b50] Stanley M. S. M. Grundmann A. W. (1970). The embryonic development of *Tribolium confusum*. Ann. Entomol. Soc. Am. 63, 1248–1256.

[b51] Strindberg H. (1915). Hauptzüge der Entwicklungsgeschichte von *Sialis lutaria* L. (Eine embryologische Untersuchung). Zool. Anz. 46, 167–185.

[b52] Tada M. Heisenberg C.-P. (2012). Convergent extension: using collective cell migration and cell intercalation to shape embryos. Development 139, 3897–3904 10.1242/dev.073007 23048180

[b53] Tiegs O. W. Murray F. V. (1938). The embryonic development of *Calandra oryzae*. Q. J. Microsc. Sci. 80, 159–273.

[b54] Toyama Y. Peralta X. G. Wells A. R. Kiehart D. P. Edwards G. S. (2008). Apoptotic force and tissue dynamics during *Drosophila* embryogenesis. Science 321, 1683–1686 10.1126/science.1157052 18802000 PMC2757114

[b55] Truckenbrodt W. (1973). Über die Entstehung der Serosa im besamten und im unbesamten Ei von *Odontotermes badius* Hav. (Insecta, Isoptera). Zeitschrift für Morphologie der Tiere 76, 193–208 10.1007/BF00280672

[b56] van der Zee M. Berns N. Roth S. (2005). Distinct functions of the *Tribolium zerknüllt* genes in serosa specification and dorsal closure. Curr. Biol. 15, 624–636 10.1016/j.cub.2005.02.057 15823534

[b57] Wakimoto B. T. Turner F. R. Kaufman T. C. (1984). Defects in embryogenesis in mutants associated with the antennapedia gene complex of *Drosophila melanogaster*. Dev. Biol. 102, 147–172 10.1016/0012-1606(84)90182-9 6421639

[b58] Wigand B. Bucher G. Klingler M. (1998). A simple whole mount technique for looking at *Tribolium* embryos. Tribolium Information Bulletin 38, 281–283.

