# Supplementary material for: High plasticity in epithelial morphogenesis during insect dorsal closure
Source: Biol Open. 2013 Sep 5;2(11):1108–18. doi: 10.1242/bio.20136072 (PMC3828757; doi:10.1242/bio.20136072)
Supplement: Supplementary Material [file supp_2_11_1108__index.html]

High plasticity in epithelial morphogenesis during insect dorsal closure — Supplementary Material 

# High plasticity in epithelial morphogenesis during insect dorsal closure

## bio.20136072 Supplementary Material

**Files in this Data Supplement:**

- Supplementary Material - Kristen A. Panfilio et al. doi: 10.1242/bio.20136072
- Movie 1 - **Movie 1. Overview of WT *Tribolium* serosal withdrawal and DC, dorsal-–lateral view, nuclear-GFP transgenic line.** Some photobleaching occurs toward the end of the movie, increasing the visibility of the dorsal yolk granules. See supplementary material Table S2 for detailed specifications.
- Movie 2 - **Movie 2. Tracked version of supplementary material Movie 1, illustrating differences between serosal and amniotic cellular reorganization.** Each nucleus is tracked in a unique color. See supplementary material Table S2 for detailed specifications.
- Movie 3 - **Movie 3. Dorsal view of WT serosal degeneration and closure of the epidermal flanks.** See supplementary material Table S2 for detailed specifications.
- Movie 4 - **Movie 4. Tracked version of supplementary material Movie 3, distinguishing the morphogenetic behavior of the serosa (blue), amnion (orange), and dorsal epidermis (green).** See supplementary material Table S2 for detailed specifications.
- Movie 5 - **Movie 5. Overview of *Tc-zen1RNAi* amniotic withdrawal and DC, lateral view.** See supplementary material Table S2 for detailed specifications.
- Movie 6 - **Movie 6. Dorsal view of *Tc-zen1RNAi* amniotic bulge migration and resorption, and closure of the epidermal flanks.** See supplementary material Table S2 for detailed specifications.
- Movie 7 - **Movie 7. Tracked version of supplementary material Movie 6, illustrating amniotic cellular reorganization.** Each nucleus is tracked in a unique color. See supplementary material Table S2 for detailed specifications.
- Movie 8 - **Movie 8. Tracked version of supplementary material Movie 6, distinguishing the morphogenetic behavior of the amnion (orange) and dorsal epidermis (green).** See supplementary material Table S2 for detailed specifications.
- Movie 9 - **Movie 9. Lateral, brightfield view of *Tc-zen1RNAi* amniotic bulge migration and resorption, and concomitant embryonic ventral flexure.** See supplementary material Table S2 for detailed specifications.
