# Supplementary material for: High plasticity in epithelial morphogenesis during insect dorsal closure
Source: Biol Open. 2013 Sep 5;2(11):1108–18. doi: 10.1242/bio.20136072 (PMC3828757; doi:10.1242/bio.20136072)
Supplement: Supplementary Material [file supp_bio.20136072_bio.20136072-s1.pdf]

Biology Open

Supplementary Material

Kristen A. Panfilio et al. doi: 10.1242/bio.20136072

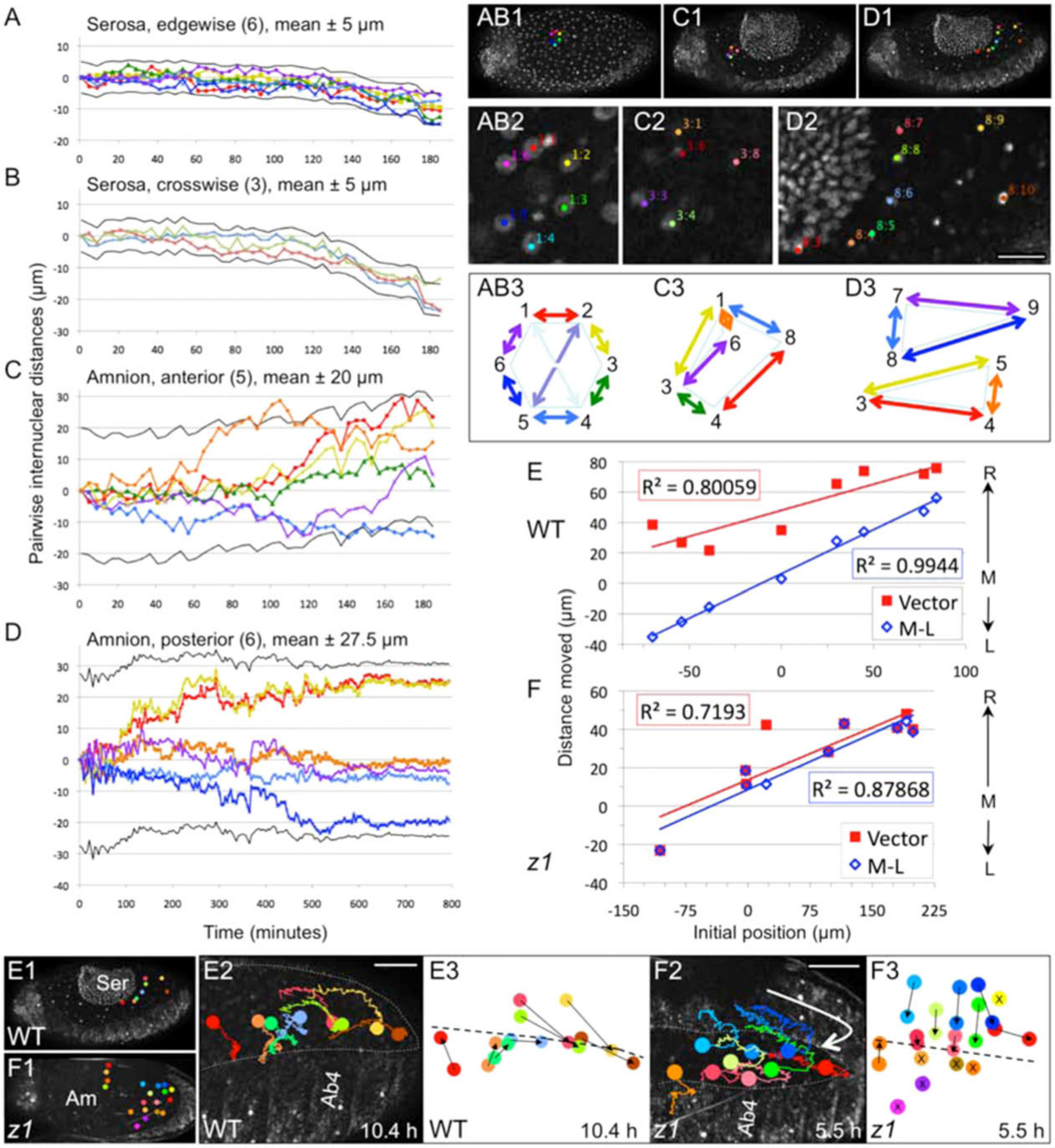

Fig. S1. See next page for legend.

Fig. S1. Differing nuclear migration behaviors within the serosa and amnion during late EE morphogenesis. (A–E) WT, (F) *Tc-zen1*^{RNAi}.
(A–D) Small clusters of 3–6 nuclei were tracked within the serosa (A,B) and amnion (C,D), during wild type serosal withdrawal and dorsal closure, showing that serosal cells move with their neighbors while amniotic cells move more individually. Data generated from supplementary material Movie 1 (supplementary material Movie 2 shows the tracked nuclei in A,B,D). Inter-nuclear distance is shown over time, where each colored line in the graph represents a given nuclear pair within the cluster. Chart titles indicate the EE tissue type, type of measurement or embryo location, number of nuclei measured, and the range encompassed by the black plot lines (mean ± offset value indicated). All distances were defined as 0 µm at time 0, such that positive and negative changes indicate increasing distance (spreading) and decreasing distance (apical contraction), respectively. When inter-nuclear distance is constant, slopes are 0. When cells within a cluster move in concert, the spread of values along the y-axis is limited. **For the serosa**, a rosette of six nuclei was measured for edgewise distances around the hexagon's circumference (A) and for crosswise distances spanning the hexagon (B). By both of these measures, inter-nuclear distance did not change appreciably (remained within 5 µm of 0) until 120 minutes (minute 64 in movie), at which time distances decreased as the serosa began to contract strongly. This conserved inter-nuclear distance was maintained as each nucleus migrated approximately 167.0±4.3 µm (mean ± standard deviation). **In the amnion**, both clusters (C,D) show marked changes in inter-nuclear distance within the first 20 minutes, as some nuclei diverge from their neighbors while others come closer together. Even though some plot lines show similar changes in distances over time (e.g. the red and yellow lines in C,D), the distances between the same nuclei and other neighbors are quite different (compare blue line in C). (E–F) Although amniotic cells do not move in concert with their neighbors, all amniotic cells migrate toward the dorsal midline, with more lateral nuclei traveling greater distances. Measurements of nuclear convergence toward the midline, showing a positive correlation between the initial distance from the midline at time 0 and the total distance moved along the medial–lateral (M–L) axis. Measurements were calculated in two ways: as a vector (length of arrows in C,D) and as simple displacement along the M–L axis so as to discount movement along the anterior–posterior axis. Positions in microns are plotted relative to the dorsal midline (=0 µm), where negative values are to the left (L) and positive values are to the right (R). Trendlines are linear. **Numbered panels** are prefaced by the letter of the corresponding graphs, where the designation AB applies to A,B. Numbering indicates: (1) whole embryo context of tracked nuclei, (2) higher magnification of selected nuclei (scale bars are 20 µm for AB2,C2,D2 and 50 µm for E2,F2), and (3) schematic representation of measured values as pairwise inter-nuclear distances (AB3,C3,D3) or as vectors of nuclear displacement (E3,F3). Numbering of nuclei merely indicates the unique identifier from tracking. Coloring in panels AB3,C3,D3 corresponds to the colors in the graphs. Panels E2,F2 are reproduced from Fig. 2F2,G3; see also supplementary material Movies 2, 7. In E3,F3, dashed lines indicate the midline; nuclei that could not be tracked for the entire interval are marked with the letter X (F3). Images are dorsal–lateral (A–E) and dorsal (F), with anterior left. Abbreviations as in main text: Am, amnion; Ab#, abdominal segment #; Ser, serosa; WT, wild type; *z1*, *Tc-zen1*^{RNAi}.

Biology Open

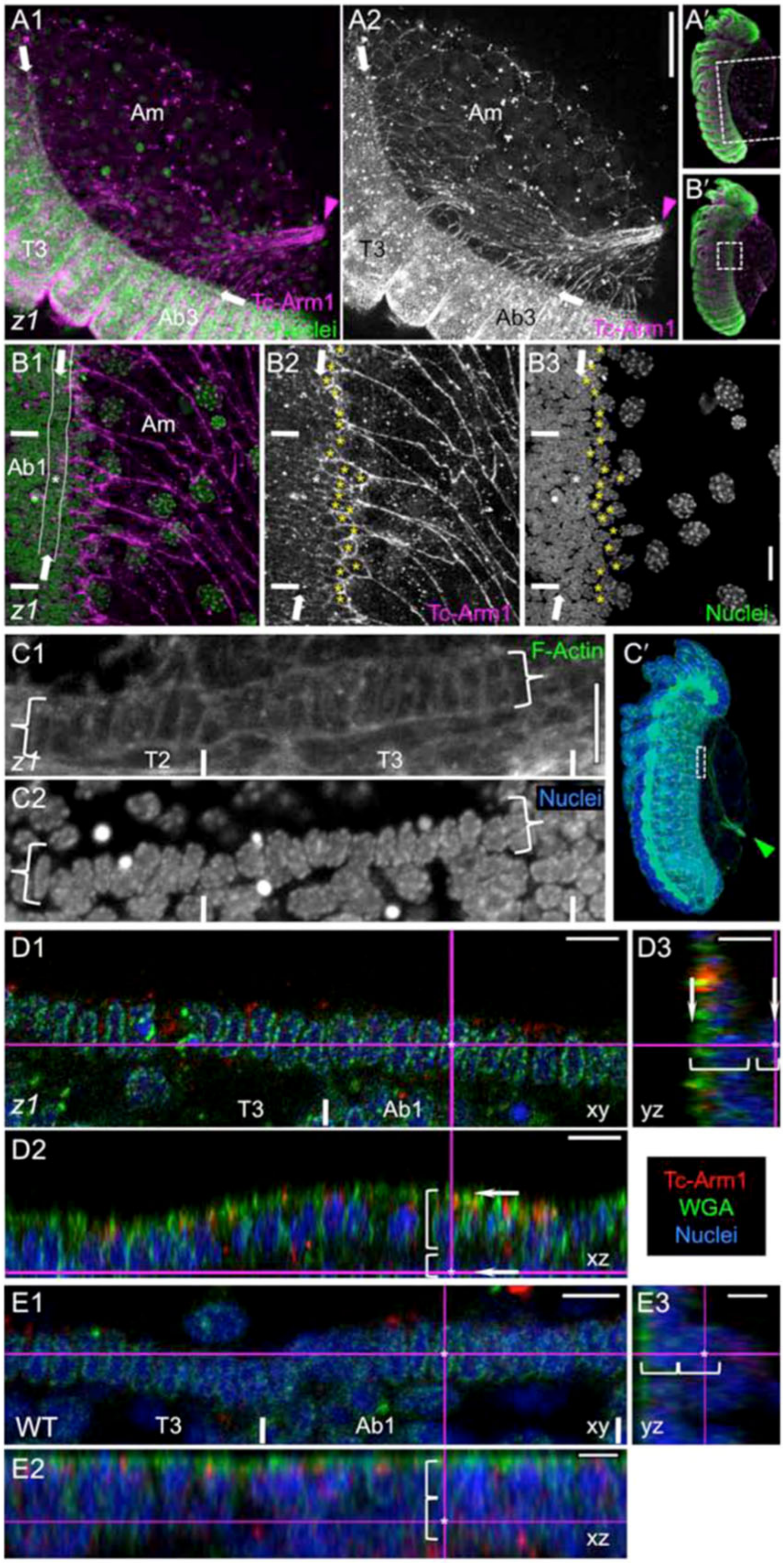

Fig. S2. Tissue structure during DC in the epidermis and amnion (as in Fig. 3). (A–D) *Tc-zen1*^{RNAi}, (E) WT. (A–B) Confocal surface projections of *Tc-zen1*^{RNAi} embryos during anteriorward migration of the amniotic crease, lateral views. The contraction and migration of the crease (arrowhead) correlate with the elongated, irregular shapes of adjacent amniotic cells. The amnion–epidermis boundary is shown at higher magnification in B1–B3. Medial–lateral (M–L) elongation of the dorsal-most epidermal cells (yellow starred cells) results in a teardrop-shaped apical area, but they do not form an orderly row. White arrows demarcate the more lateral, mesodermal cardioblast cell row (also partially outlined with white lines in B1). The white asterisk in B1–B3 marks the position of the selected cell shown in D, below. (C–D) The cardioblast cell row, in contrast, is clearly organized. As in WT, it consists of a single cell row that spans the length of the flank, with both medial and lateral supracellular F-Actin cables (C: curly brackets; compare with WT in Fig. 3E). However, it is not apparent in surface projections (B) as it is subepidermal. Images C1,C2 show a subsurface, partial projection (6.75 µm thickness). Images D1–D3 are single optical sections, focusing on the white starred cell in B: D1, xy plane (anterior–posterior (A–P) and dorsal–ventral (D–V)); D2, xz plane (A–P and apical–basal); D3, yz plane (D–V and apical–basal). In D2 and D3, white brackets in the apical–basal plane demarcate the selected cell (partially) and the overlying epidermal cell; D1 is an optical slice 7 µm below the apical surface of the epidermis. (E) The cardioblast cell row also has this subepidermal position in WT (same embryo as in Fig. 3A), although in later DC it is more apparent in surface views in both WT and *Tc-zen1*^{RNAi} embryos (Fig. 5 and data not shown). All images are in lateral aspect, with anterior up/left and dorsal right/up. In whole embryo views (letter-prime panels), regions of interest are indicated by dashed boxes (B′ applies to both B and D). Fluorescent staining reagents are as indicated (Arm1 is anti-Tc-Arm1). Abbreviations as above, additionally: T#, thoracic segment #. Scale bars: 50 µm (A1,A2), 10 µm (B1–B3), and 5 µm (C1,C2, D1–D3, E1–E3).

Biology Open

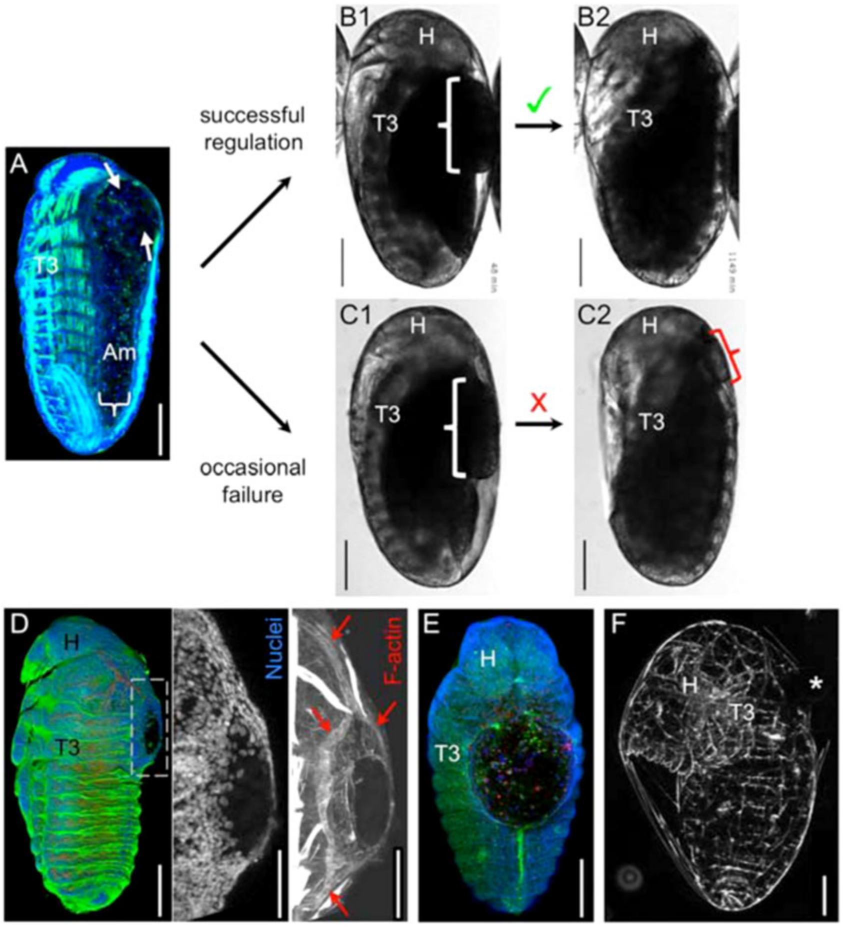

Fig. S3. Occasional failure of final DC in *Tc-zen1*^{RNAi} embryos. DC is less robust in *Tc-zen1*^{RNAi} embryos than in WT. Periodically the embryos fail to resorb the anterior amniotic/yolk bulge, leading to anterior dorsal defects that range in severity. (A) Dorsal–lateral micrograph of a Tc-zen1^{RNAi} embryo at mid-DC stage, with white arrows labeling handling damage in the anterior bulge region (reproduced from Fig. 1M: green: F-Actin, blue: nuclei). (B–C) Brightfield, transmitted light still images in lateral aspect. In B1,B2, the anterior bulge (white bracket) is successfully resorbed (stills from supplementary material Movie 9, corresponding to Fig. 6I). In contrast, C1,C2 show a small quantity of yolk that ends up outside of the closed dorsal epidermis (red bracket in C2), from a second embryo recorded simultaneously and on the same slide as the embryo shown in B. (D–E) Confocal projections in dorsal–lateral (D) and dorsal (E) aspect, illustrating the varying sizes of lesions when DC is further disturbed and the left and right epidermal flanks fail to close in the anterior region of the dorsum. Note that the defects affect the thorax (and abdominal) segments, whereas closure of the head capsule is unaffected. In the higher magnification images in D (corresponding to the boxed region in the whole mount view), red arrows highlight the left and right cardioblast cell rows, which had successfully merged at the midline anterior and posterior to the lesion. Staining reagents are WGA (green), nuclei (blue), F-Actin (red in D), and the caspase apoptosis marker (red in E). (F) Cuticle preparation in ventral–lateral aspect at hatching stage. The embryo has elongated and the head is tucked down onto the legs, stretching the vitelline membrane (eggshell component). This embryo also has a dorsal lesion in the thoracic region, marked with an asterisk. Cuticle preparations essentially followed existing protocols (van der Zee et al., 2005). Anterior is up in all images, and dorsal is right in lateral views. Scale bars: 100 µm for all whole mounts, 50 µm for the higher magnification views in D.

Table S1. Numerical description of final DC scalloping closure for three stage-matched *Tribolium* embryos for each of wild type (WT) and *Tc-zen1*^{RNAi} (*z1*) treatments. (A) These values indicate by which criteria and how closely the WT and *Tc-zen1*^{RNAi} embryos were morphologically stage-matched for "young", "mid", and "old" stages. Measurements were made in ImageJ on micrographs of maximum intensity confocal projections of whole-mount specimens stained for F-Actin and nuclei. (B–D) For most features, there is no difference between WT and *Tc-zen1*^{RNAi} embryos. Statistical tests in D are paired Student's *t*-Tests (http://www.physics.csbsju.edu/stats/t-test.html; last accessed 11 December 2012). (E) However, the difference in closure geometry between WT and *Tc-zen1*^{RNAi} embryos is reflected in which regions along the anterior–posterior axis are more widely open for a given stage: WT embryos are most closed in the thorax, while *Tc-zen1*^{RNAi} embryos are most open in this body tagma. See also Fig. 5.

Feature	WT	*z1*
**A. Open dorsum area (# segments: segment identity, linear length (μm))**		
Young	9: T1-Ab6, 452.21 μm	11: T1-Ab8, 495.52 μm
Mid	9: T1-Ab6, 421.13 μm	9: T1-Ab6, 383.67 μm
Old	7: T2-Ab5, 350.04 μm	11: T1-Ab8, 369.44 μm
**B. Degree of scalloping (# paired scalloped maxima, # unpaired and which flank)**		
Young	11 paired, 0 unpaired	10 paired, 0 unpaired
Mid	8 paired, 1 unpaired (left)	10 paired, 2 unpaired (left)
Old	9 paired, 0 unpaired	9 paired, 1 unpaired (right)
**C. Deviations from 1 pair of scalloped maxima/body segment**		
Young	2 in each of T1 and T2, none clearly in Ab6	0 in T1 (bulge), 2 in Ab3, 0 in Ab8
Mid	0 in Ab6	2 in T1
Old	2 in T2, 3 in T3, 2 in Ab1	0 in T1 (closing), 2 in T2, 0 in Ab1, 0 in Ab8 (closing)
**D. Degree of epidermal edge "wiggliness" due to scalloping *vs* a smooth curve**		
D1. Length ratio ((actual edge)/(smooth curve)): left flank, right flank		
Young	1.0495, 1.0632	1.0804, 1.0270
Mid	1.0744, 1.0273	1.1104, 1.1100
Old	1.1012, 1.1103	1.0366, 1.1034
D2. All stages, both flanks: mean ± standard deviation	1.071±0.031	1.078±0.038
D3. Actual edge longer than smooth curve length	Significant (t=5.55, degrees of freedom = 5, P=0.003)	Significant (t=5.09, degrees of freedom = 5, P=0.004)
D4. Degree of symmetry (length ratios of left *vs* right flanks)	No significant difference (t=0.125, degrees of freedom = 2, P=0.912)	No significant difference (t=−0.414, degrees of freedom = 2, P=0.719)
**E. Open medial distance between paired scalloped maxima (μm, body segment ID)**		
E1. Maximum: Young	66.40 μm (Ab3)	123.78 μm (T2: anterior amniotic bulge)
Mid	43.09 μm (Ab5)	55.81 μm (Ab4)
Old	33.68 μm (Ab3)	58.12 μm (T2)
E2. Minimum: Young	36.52 μm (T1)	42.42 μm (Ab7)
Mid	8.73 μm (T1)	11.15 μm (T1: nearly closed anterior end)
Old	10.70 μm (T3)	22.28 μm (Ab7)

Biology Open

Table S2. Time-lapse movie specifications. Time stamps (minutes) are relative to the start of the movie, except in supplementary material Movie 1, where time is relative to serosal rupture at t=0. The autofluorescent vitelline membrane can be seen in all films. Versions with nuclear tracking have the same identifying number as the unlabeled version, and are distinguished by letter. Track colors are the same as in the corresponding figures, where colors distinguish individual cells (supplementary material Movies 2, 7), or tissue types (supplementary material Movies 4, 8). All files play at 8 frames per second. Anterior is left, and dorsal is up for lateral views. All scale bars are 100 µm.

**Movies**	**1, 2**	**3, 4**	**5**	**6, 7, 8**	**9**
Treatment	WT	WT	*Tc-zen1*^{RNAi}	*Tc-zen1*^{RNAi}	*Tc-zen1*^{RNAi}
Angle of view	Dorsal–lateral	Dorsal	Lateral	Dorsal	Lateral
Illumination type	GFP (ubiquitous nuclear-GFP transgenic line)	GFP (ubiquitous nuclear-GFP transgenic line)	GFP (ubiquitous nuclear-GFP transgenic line)	GFP (ubiquitous nuclear-GFP transgenic line)	Transmitted white light
Developmental stages shown	56 min prior to serosal rupture through very late serosal degeneration during late DC	Early serosal degeneration stage to late DC	Early amniotic "crease stage" to completion of ventral postural flexure in late DC	Early amniotic "crease stage" to late DC	Amniotic "anterior bulge" stage to completion of ventral flexure, then post-DC (after a 7.1-hour gap)
Corresponding figure	Fig. 1, overview; Fig. 2E–F (supplementary material Movie 2)	Fig. 4, overview; Fig. 4A–B (supplementary material Movie 4)	Fig. 1, general overview	Fig. 2, overview; Fig. 2G (supplementary material Movie 7); Fig. 4C–E (supplementary material Movie 8)	Fig. 6B
Recording temperature (℃)	25℃	25℃	25℃	25℃	22℃
Recording interval frequency	Every 5 min	Every 5 min	Every 8 min	Every 5 min (first 530 min), then every 10 min	Every 1 min (first 70 min), then every 2 min
Movie play rate	2100 × (35 min/sec)	2100 × (35 min/sec)	3360 × (56 min/sec)	2100 to 4200 × (35 to 70 min/sec)	60 to 120 × (1 to 2 min/sec)
Projection? (step size/ stack size)	Yes, 5/65 µm, max. intensity	Yes, 5/55 µm, max. intensity	Yes, 5/40 µm, max. intensity	Yes, 5/50 µm, max. intensity	No, single focal plane

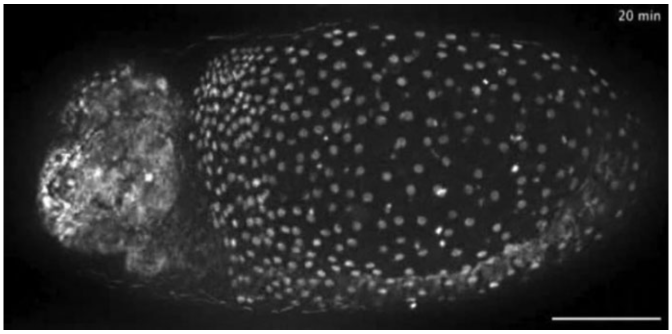

Movie 1. Overview of WT *Tribolium* serosal withdrawal and DC, dorsal–lateral view, nuclear-GFP transgenic line. Some photobleaching occurs toward the end of the movie, increasing the visibility of the dorsal yolk granules. See supplementary material Table S2 for detailed specifications.

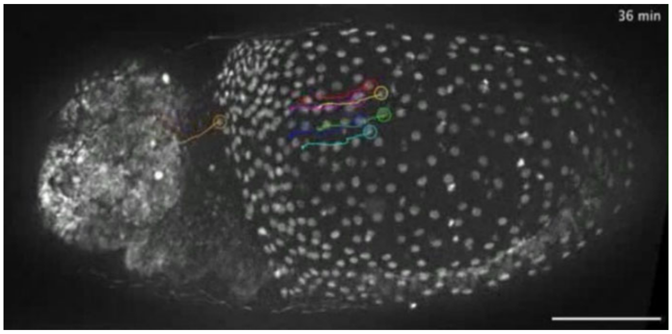

Movie 2. Tracked version of supplementary material Movie 1, illustrating differences between serosal and amniotic cellular reorganization. Each nucleus is tracked in a unique color. See supplementary material Table S2 for detailed specifications.

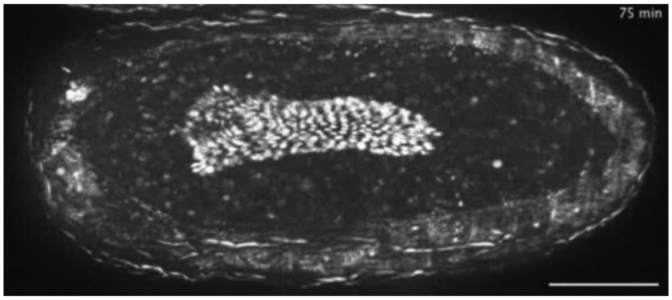

Movie 3. Dorsal view of WT serosal degeneration and closure of the epidermal flanks. See supplementary material Table S2 for detailed specifications.

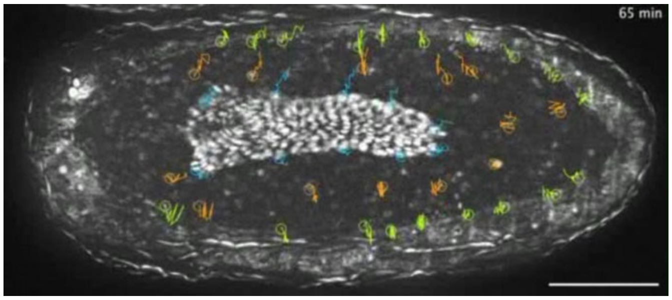

Movie 4. Tracked version of supplementary material Movie 3, distinguishing the morphogenetic behavior of the serosa (blue), amnion (orange), and dorsal epidermis (green). See supplementary material Table S2 for detailed specifications.

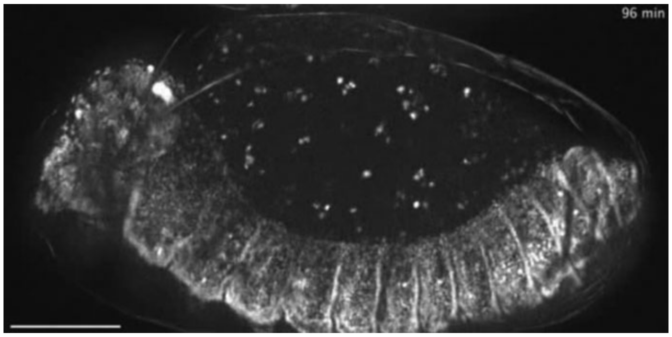

Movie 5. Overview of *Tc-zen1*^{RNAi} amniotic withdrawal and DC, lateral view. See supplementary material Table S2 for detailed specifications.

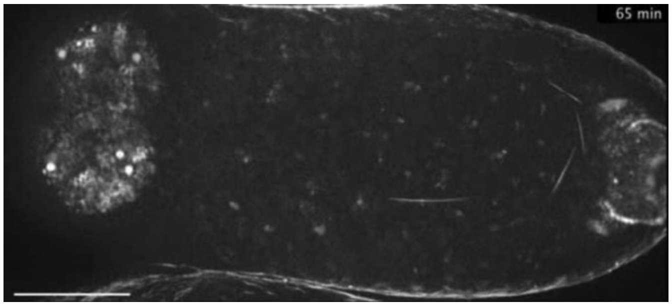

Movie 6. Dorsal view of *Tc-zen1*^{RNAi} amniotic bulge migration and resorption, and closure of the epidermal flanks. See supplementary material Table S2 for detailed specifications.

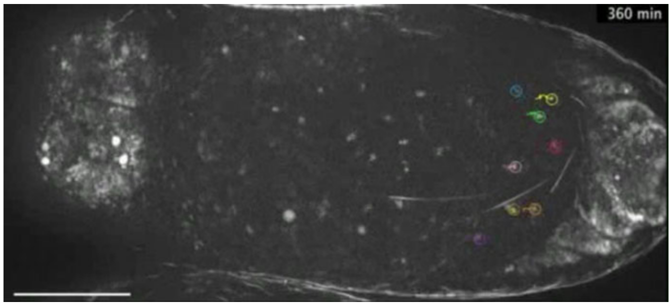

Movie 7. Tracked version of supplementary material Movie 6, illustrating amniotic cellular reorganization. Each nucleus is tracked in a unique color. See supplementary material Table S2 for detailed specifications.

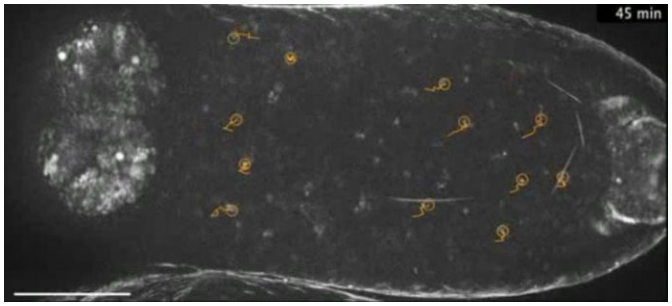

Movie 8. Tracked version of supplementary material Movie 6, distinguishing the morphogenetic behavior of the amnion (orange) and dorsal epidermis (green). See supplementary material Table S2 for detailed specifications.

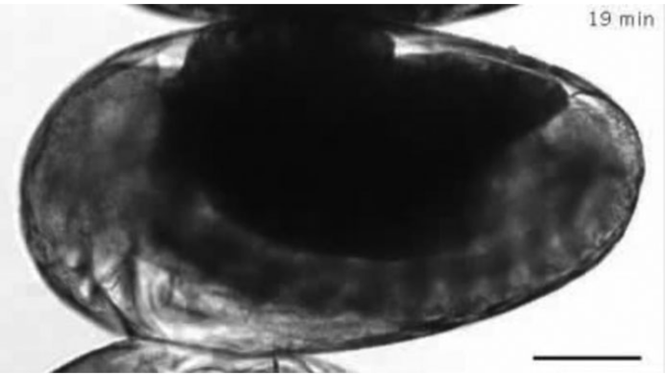

Movie 9. Lateral, brightfield view of *Tc-zen1*^{RNAi} amniotic bulge migration and resorption, and concomitant embryonic ventral flexure. See supplementary material Table S2 for detailed specifications.

Biology Open
